# Finding a Novel Chalcone–Cinnamic Acid Chimeric Compound with Antiproliferative Activity against MCF-7 Cell Line Using a Free-Wilson Type Approach

**DOI:** 10.3390/molecules28145486

**Published:** 2023-07-18

**Authors:** Isis A. Y. Ventura-Salazar, Francisco J. Palacios-Can, Leticia González-Maya, Jessica Nayelli Sánchez-Carranza, Mayra Antunez-Mojica, Rodrigo Said Razo-Hernández, Laura Alvarez

**Affiliations:** 1 Centro de Investigaciones Químicas, Instituto de Investigación en Ciencias Básicas y Aplicadas, Universidad Autónoma del Estado de Morelos, Av. Universidad No. 1001, Cuernavaca 62210, Mexico; isis.venturaz@uaem.edu.mx; 2 Centro de Investigación en Dinámica Celular, Instituto de Investigación en Ciencias Básicas y Aplicadas, Universidad Autónoma del Estado de Morelos, Av. Universidad No. 1001, Cuernavaca 62210, Mexico; fjpc@uaem.mx; 3 Facultad de Farmacia, Universidad Autónoma del Estado de Morelos, Av. Universidad No. 1001, Cuernavaca 62210, Mexico; letymaya@uaem.mx (L.G.-M.); jessica.sanchez@uaem.mx (J.N.S.-C.); 4 CONAHCYT-Instituto de Investigación en Ciencias Básicas y Aplicadas, Centro de Investigaciones Químicas, Universidad Autónoma del Estado de Morelos, Av. Universidad No. 1001, Cuernavaca 62210, Mexico; myam@uaem.mx

**Keywords:** chalcone, cytotoxic activity, hybrid molecule, QSAR

## Abstract

In this work, we carried out the design and synthesis of new chimeric compounds from the natural cytotoxic chalcone 2′,4′-dihydroxychalcone (2′,4′-DHC, **A**) in combination with cinnamic acids. For this purpose, a descriptive and predictive quantitative structure–activity relationship (QSAR) model was developed to study the chimeric compounds’ anti-cancer activities against human breast cancer MCF-7, relying on the presence or absence of structural motifs in the chalcone structure, like in a Free-Wilson approach. For this, we used 207 chalcone derivatives with a great variety of structural modifications over the α and β rings, such as halogens (F, Cl, and Br), heterocyclic rings (piperazine, piperidine, pyridine, etc.), and hydroxyl and methoxy groups. The multilinear equation was obtained by the genetic algorithm technique, using logIC_50_ as a dependent variable and molecular descriptors (constitutional, topological, functional group count, atom-centered fragments, and molecular properties) as independent variables, with acceptable statistical parameter values ($R^{2}$ = 86.93, $Q^{2}$*_LMO_* = 82.578, $Q^{2}$*_BOOT_* = 80.436, and $Q^{2}$*_EXT_* = 80.226), which supports the predictive ability of the model. Considering the aromatic and planar nature of the chalcone and cinnamic acid cores, a structural-specific QSAR model was developed by incorporating geometrical descriptors into the previous general QSAR model, again, with acceptable parameters ($R^{2}$ = 85.554, $Q^{2}$*_LMO_* = 80.534, $Q^{2}$*_BOOT_* = 78.186, and $Q^{2}$*_EXT_* = 79.41). Employing this new QSAR model over the natural parent chalcone 2′,4′-DHC (**A**) and the chimeric compound 2′-hydroxy,4′-cinnamate chalcone (**B**), the predicted cytotoxic activity was achieved with values of 55.95 and 17.86 µM, respectively. Therefore, to corroborate the predicted cytotoxic activity compounds **A** and **B** were synthesized by two- and three-step reactions. The structures were confirmed by ^1^H and ^13^C NMR and ESI+MS analysis and further evaluated in vitro against HepG2, Hep3B (liver), A-549 (lung), MCF-7 (breast), and CasKi (cervical) human cancer cell lines. The results showed IC_50_ values of 11.89, 10.27, 56.75, 14.86, and 29.72 µM, respectively, for the chimeric cinnamate chalcone **B**. Finally, we employed **B** as a molecular scaffold for the generation of cinnamate candidates (**C**–**K**), which incorporated structural motifs that enhance the cytotoxic activity (pyridine ring, halogens, and methoxy groups) according to our QSAR model. ADME/tox in silico analysis showed that the synthesized compounds **A** and **B**, as well as the proposed chalcones **C** and **G**, are the best candidates with adequate drug-likeness properties. From all these results, we propose **B** (as a molecular scaffold) and our two QSAR models as reliable tools for the generation of anti-cancer compounds over the MCF-7 cell line.

## 1. Introduction

“Cancer” is a generic term that designates a broad group of diseases that can affect any part of the body [1]. It is a tissue growth produced by the continuous proliferation of abnormal cells with the ability to invade and destroy other tissues. It is a consequence of gene mutations that control cell proliferation, differentiation, and homeostasis [2]. The International Agency for Research on Cancer estimated 18.1 million new cancer cases and 10 million cancer deaths in 2020 [3,4]. In México, 190,667 new cases of cancer were presented in 2018, including breast, prostate, colorectal, thyroid, and cervical cancers [4]. Currently, the available treatments for cancer patients can be classified into those that are applied locally (such as surgery and radiotherapy) and those that are used systemically (chemotherapy, hormone therapy, and immunotherapy). The search for suitable natural products for treating human cancer has resulted in the discovery of powerful antiproliferative chemicals used in current practice. Molecular models (scaffolds) obtained from living organisms have undergone different modifications to obtain molecules that are more potent and less toxic than the original molecules.

Chalcone is a simple chemical scaffold of a wide variety of natural and synthetic flavonoid-type compounds. They are widely distributed in the vegetable kingdom, being present in vegetables, fruits, teas, and other plants [5,6]. Chalcone derives from the Greek word “Chalcos”, meaning “bronze”, which results from the colors of most natural chalcones [7]. Structurally, chalcones consist of a common chemical scaffold of 1,3-diaryl-2-propen-1-one (chalconoid), which is formed by two aryl groups (α- and β-rings) connected by an enone, existing as cis and trans isomers, with the trans isomer being thermodynamically more stable. In general, chalcones are synthesized under Claisen–Schmidt condensation conditions using acetophenone and substituted benzaldehydes [8,9,10]. In order to obtain condensate compounds, in the case of polyhydroxy chalcones, protection/deprotection steps are employed [11,12,13,14].

Research shows that chalcones have a wide and interesting variety of biological activities, including cytotoxic, antioxidant, anti-cancer, antimicrobial, antiviral, antiprotozoal, antiulcer, anti-inflammatory, antihistaminic, and antidepressant activities [15]. As for anti-tumor activity, chalcones exhibit various effects, including anti-initiation, apoptosis induction, antiproliferation, antimetastasis, antiangiogenesis, DNA and mitochondrial damage, tubulin inhibition, etc. [16,17]. Among the naturally occurring hydroxy chalcones, 2′,4′-dihydroxychalcone (**A**), which is a flavonoid isolated from various medicinal plants [18,19], displays antibacterial cytotoxic and antifungal properties [20,21]. Experimental studies show the antiproliferative activity of 2′,4′-dihydroxychalcone on cancer cells. Moreover, it is demonstrated to induce apoptosis in human gastric cancer cells [22], damage to the DNA results in a Rad3-dependent and Chk1-dependent cell cycle, and blocks at the G2/M transition [22,23].

Other naturally occurring compounds with a wide variety of biological properties are phenolic acids, vital constituents found in plants, fruits, vegetables, cereals, and legumes [24]. As secondary metabolites produced by plants, phenolic compounds are characterized by a wide variety of biological properties, with standouts including antioxidant, anti-inflammatory, hepatoprotective, anxiolytic, insect-repellent, antidiabetic, anticholesterolemic, antimicrobial, and antiulcer activities [25]. In terms of chemical structure, they can be divided into derivatives of cinnamic and benzoic acids, varying in the number and substitution of hydroxyl and methoxy groups. Cinnamic, ferulic, caffeic, and *p*-coumaric acids are the most common cinnamic acid derivatives, and gallic, protocatechuic, syringic, salicylic, vanillic, and *p*-hydroxybenzoic acids are the most common benzoic acid derivatives [26]. Cinnamic acid is a key intermediate in the shikimate and phenylpropanoid pathways [27]. Cinnamic acid derivatives are reported to have strong antioxidant effects [28]. The *p*-hydroxy and methoxy groups in cinnamic acid derivatives also show good insulin-releasing activity [29]. Benzoic acid derivatives are naturally consumed as dietary phenolic compounds.

On the border between bioinspired design and rational design, we can prepare hybrid molecules with a dual mode of action to create new efficient drugs. Hybrid molecules are defined as chemical entities with two or more structural domains with different biological functions and double activity, indicating that a hybrid molecule acts as two distinct pharmacophores [30]. For these reasons, as well as their structural simplicity and the simplicity of their associated synthesis, chalcones and polyphenols continue to enjoy considerable attention from medicinal chemicals that explore new molecular scaffolds for the design of new therapeutics [31].

The use of in silico techniques, such as quantitative structure–activity relationship studies (QSAR), in pharmacology has improved the process of selection and design of several drugs. All these attributes originated the Computer-Aided Drug Design (CADD) discipline. QSAR is a powerful tool to discover the underlying relationship between a molecular structure and the biological activity of a set of compounds, supported by rigorous statistical parameters [32,33,34,35]. Many QSARs have been done to study the biological activities of the chalcone and polyphenols compounds; our work studied the possible action mechanism of a particular set of chalcones with antiproliferative activity [20]. In addition, QSAR is a valuable tool for achieving the prediction of activity in cases where experimental data are not accessible. QSAR methods use mathematical functions to relate the molecular characteristics and properties of the compounds with their biological activity.

Free-Wilson analysis is a QSAR method that directly relates the structural features of molecules with their biological properties, offering a simple and efficient approach for the quantitative description of the biological activity of interest. The main advantage of employing this methodology is the use of simple descriptors that consider only the presence or absence of substituents at a specific position on the molecule–molecular scaffold. However, some drawbacks arise, mainly due to the fact that a large number of analogs need to be synthesized to make the model meaningful.

Based on all the properties mentioned above of the chalcone and phenolic compounds, in this work, we constructed a QSAR model to predict the cytotoxic activity of different 2′,4′-DHC derivatives replaced in position C-4′ with phenols from the cinnamic and benzoic acid families. Finally, we synthesized and evaluated the theoretically more active compound in a panel of five cell lines of human cancer.

## 2. Results

### 2.1. QSAR Study

**Figure 1:**
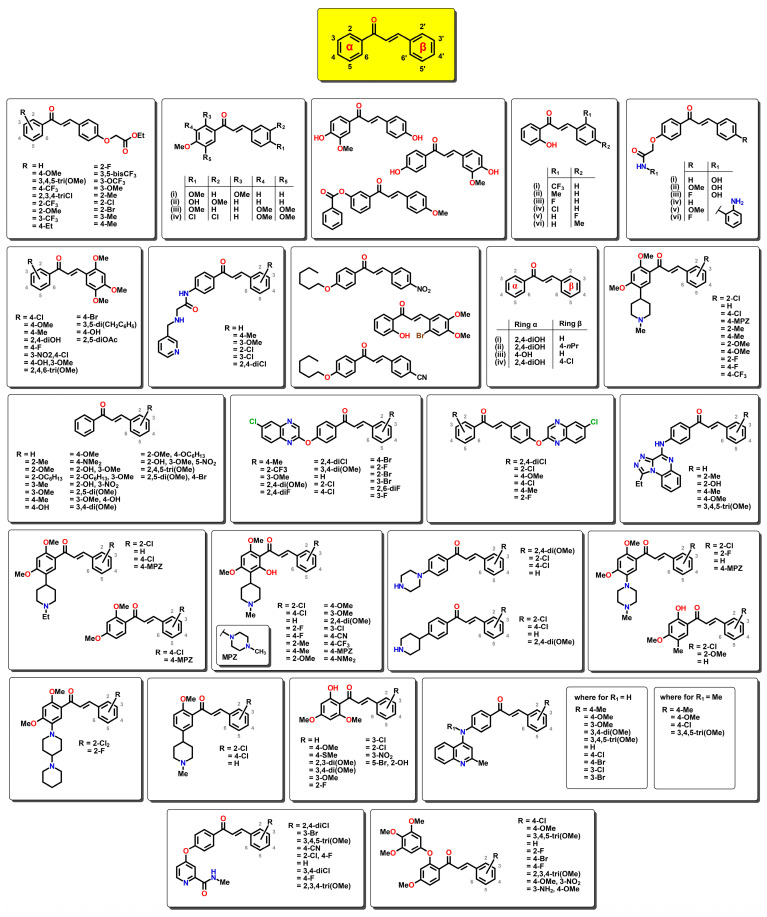
Structure of chalcone derivatives used in the generation of the QSAR model. General numbering at rings α and β in the chalcone template used for the generation of molecular descriptors are seen at the top of the figure.

A set of 207 molecules with cytotoxic activity against breast cancer line MCF-7 was selected from the literature [36,37,38,39,40,41,42,43,44,45,46,47,48,49,50,51,52,53]. Molecules express a variable range in their half-maximal inhibitory concentration (IC_50_); see Table S1. The criterion of selection of molecules was based on the structural variability around the chalcone core (small, medium, and large substituents on rings “α” and “β”, Figure 1), as well as being evaluated in the same cell line. Logarithmic transformation of IC_50_ values was achieved to normalize the experimental information.

The calculation of different descriptor families was based on their structural interpretation to help us to find the structural fragments that are key for increasing chalcone antiproliferative activity (see details in the Computational methods section). A Free-Wilson analysis was done by inspection of substituents at rings α and β: if a specific functional group appeared at a particular position in the ring, a number 1 was assigned; otherwise, a 0 value was designated. The generation of mathematical models was done through the MobyDigs software [54]; genetic algorithms (GA) were used to generate regression models. The best mathematical model obtained is shown below, consisting of fifteen molecular descriptors. (Equation (1)).

logIC_50_ = (0.3774 ± 0.0016)**R6_OH** + (−0.3463 ± 0.0003)**R2_OMe** + (−1.1201 ± 0.0014)**R4_FA026** + (0.3597 ± 0.0078)**R4_FA029** + (0.5603 ± 0.0011)**R4_1PPD** + (−0.56 ± 0.0004)**R2_TMPhO** + (−0.0736 ± 0.0006)**R2′_Cl** + (−0.2414 ± 0.0034)**R4′_Cl** + (0.0941 ± 0.0002)**R2′_OMe** + (0.3883 ± 0.0009)**R4′_4MPZ** + (−0.0824 ± 0.0014)**R4′_FB035** + (−0.0847 ± 0.0000)**Qindex** + (0.0003 ± 0.0000)**CENT** + (0.4083 ± 0.0074)**C029** + (−0.0378 ± 0.0000)**H052** + 2.2688 ± 0.0042 (1)

$$n=160;\;R^{2}=86.93\;(\pm 1.3891);\;{R^{2}}_{\mathrm{ADJ}}=84.888\;(\pm 1.8627);\;a(R^{2})=0.1026\;(\pm 0.0000);\;\;s=0.1501\;(\pm 0.0000);\;F=42.906\;(\pm 22.1114);\;{Q^{2}}_{\mathrm{LMO}}=82.578\;(\pm 2.3783);\;\;{Q^{2}}_{\mathrm{BOOT}}=80.436\;(\pm 2.9256);\;{Q^{2}}_{\mathrm{EXT}}=80.226\;(\pm 19.0111);\;a(Q^{2})=-0.2392\;(\pm 0.000);\;\;\delta K=0.0308\;(0.000);\;\delta Q=0.0034\;(-0.005);\;R^{P}=0.017\;(0.100);\;R^{N}=-0.033\;(-0.054)$$

**Figure 2:**
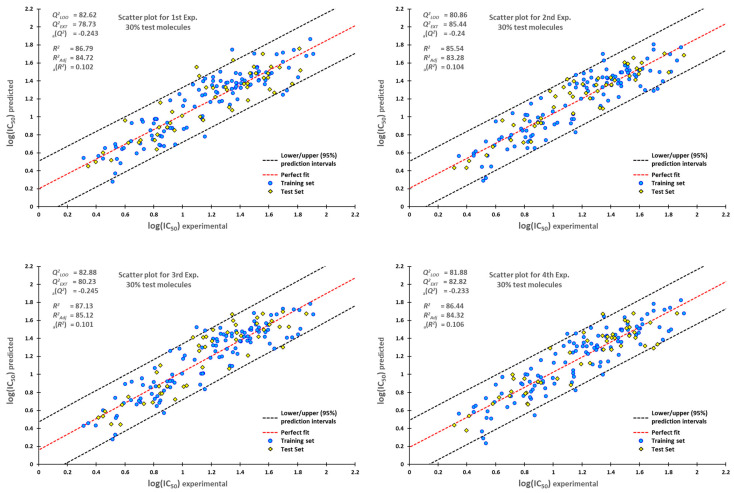
Scatterplots of predicted logIC_50_ against experimental logIC_50_ values. Blue dots represent molecules of the training set (70%), and yellow diamonds depict molecules used for the test set (30%).

All statistical parameters were obtained as an average value from five experiments. The values of all the molecular descriptors present in the model and the logIC_50_ of each molecule are shown in Table S1. This model was chosen according to its descriptive ability—associated with the molecular descriptors and their coefficients—and statistical validation requirements, such as standard deviation (s) of 0.1501 (±0.000) [55], Fisher function test (F) value of 42.906 (±22.1114) [56], and correlation coefficient ($R^{2}$) of 86.93 (±1.3891) [57], indicating that our model is acceptable. In addition, the QUIK (δK), redundancy ($R^{P}$), and overfitting ($R^{N}$) rules were used for the model selection with values of δK = 0.0308 (0.000), $R^{P}$ = 0.017 (0.100), and $R^{N}$ = −0.033 (−0.054) [58,59,60,61], where $R^{P}$ indicates redundancy among some descriptors. To evaluate and validate the predictive ability of our model, we applied the asymptotic squared Q rule ($Q^{2}$*_ASYM_*) and the leave-many-out cross-validation ($Q^{2}$*_LMO_*), this technique consists of leaving a percentage of the molecules out of the model generation; in our case, it was 30% randomly selected, and the experiment was carried out five times. Linear correlation plots of the predicted logIC_50_ against experimental logIC_50_ are shown in Figure 2, with their upper/lower confidence intervals at a 95% level [61]. From Figure 2, it can be seen the correlation and predictive ability of our QSAR model according to the number of molecules (colored blue and yellow) that are located in the applicability domain.

**Figure 3:**
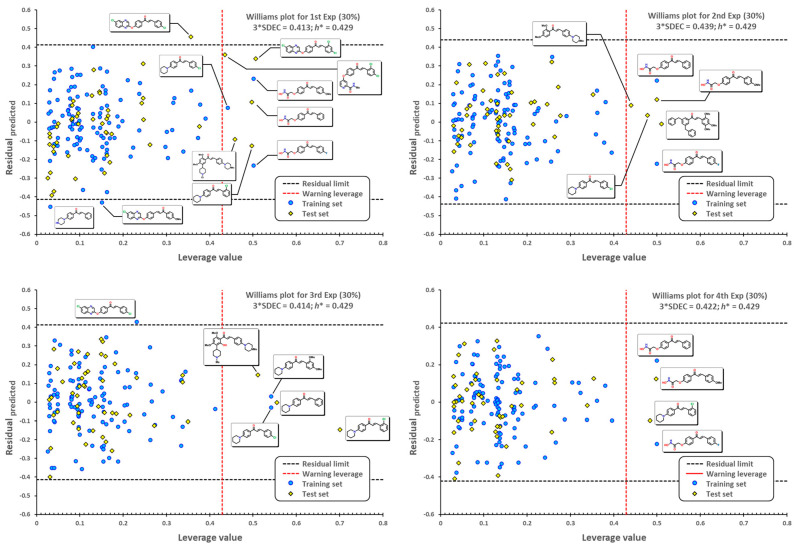
Williams’ plots for molecules with cytotoxic activity. The dotted vertical line in red indicates the warning leverage limit. The upper/lower dotted horizontal lines in black represent the boundaries for which the triple of the standard deviation (3*SDEC) value is used.

The applicability domain depicted by the Williams plot in Figure 3 allows the detection of molecules that our model cannot adequately predict. Molecules with distinctive structures—high leverage, *h* > h*, where h* = (3p)/n, where n is the number of molecules and p is the number of descriptors in the model plus one—or those associated with the response (calculated residuals > 3*SDEC), that are outside of the limits established by the leverage warning and three times the standard deviation in error calculation are outliers.

Three chalcones containing the 6-chloro-2-quinoxalinol motif are seen as outliers, with two containing chlorine atoms at positions 2 and 4 on the β-ring and one *p*-methoxy group on the α-ring. Although the QSAR model suggests that chlorine atoms located at the *ortho*- and *para*-positions on the β-ring are good for the bioactivity, the combination with the 6-chloro-2-quinoxalinol group generates results that are out of our model applicability domain for a bit. The *p*-methoxy is distinctive from the rest of the molecules in terms of bioactivity: while similar species do exhibit logIC_50_ below 1.35 (or 22 µM), the methoxy derivative shows a logIC_50_ value of 1.725 (or 53.1 µM), thus being less potent than other compounds. Hydroxamic acid derivatives are also observed as outliers, as only three molecules are present within the data used to generate the model. Piperidine derivatives, alongside a piperazine chalcone, are also observed as outliers. Indeed, Equation (1) establishes that piperidine substituents at the α-ring decrease the chalcones’ potency. Other compounds with unique structures are observed in Figure 3, for which William’s plots placed them as potential outliers.

### 2.2. QSAR Interpretation

Equation (1) presents eleven indicative variables: R6_OH, R2_OMe, R4_FA026, R4_FA029, R4_1PPD, R2_TMPhO, R2′_Cl, R4′_Cl, R2′_OMe, R4′_4MPZ, and R4′_FB035. These descriptors are associated with the presence/absence of the corresponding functional group placed at the specific carbon atom on rings “α” or “β”. Functional groups are important features in chemical structures as they provide the basis for physicochemical properties unique to each compound, as well as reactivity. They also allow the generation of key interactions with their biological targets and can modulate the potency of affinity. Thus, the inclusion of molecular descriptors of functional groups at specific positions in the chalcones rings permits the analysis of their potency in terms of volume or properties. Nevertheless, for the study of this system, the consideration of common functional groups as molecular fragments was not enough. Therefore, bigger and more complex molecular fragments incorporated on rings “α” or “β” were used.

**Figure 4:**
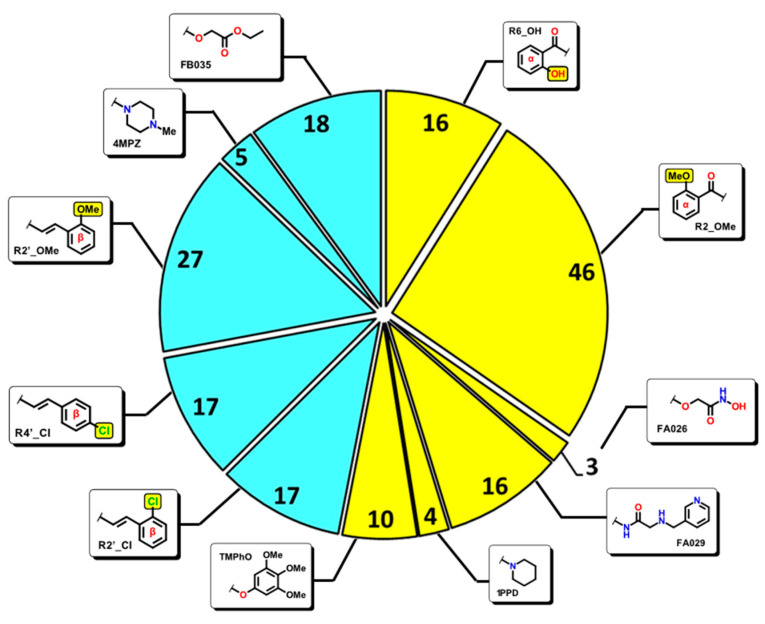
Structure of molecular fragments used as indicative variables: R6_OH, R2_OMe, FA026, FA029, TMPhO, and 1PPD belongs to α-ring (highlighted in yellow), while R2′_Cl, R4′_Cl, R2′_OMe, R4′_4MPZ, and R4′_FB035 belong to the β-ring (highlighted in cyan).

Figure 4 shows the structure of functional groups and structural fragments used in the model and the number of molecules from the dataset that contain it. For example, it is observed that 16 molecules from the 207 compounds contain the hydroxyl group at the α-ring, while 46 contain the methoxy group on the same ring. On the other hand, 17 molecules from the 207 chalcones contain the chlorine atom at position 2, while another 17 molecules contain the Cl-atom at position 4 of the β-ring. A reduced number of chalcones contain complex molecular fragments, as in most cases, these molecular descriptors are used to incorporate this diverse set of molecules into the QSAR model. For example, according to the Williams’ plots, the chemical structure FA026 only appears in three of the 207 molecules, which correlates with their position as chemical outliers. The same applies to molecules that contain the 4MPZ and 1PPD functional groups.

**Figure 5:**
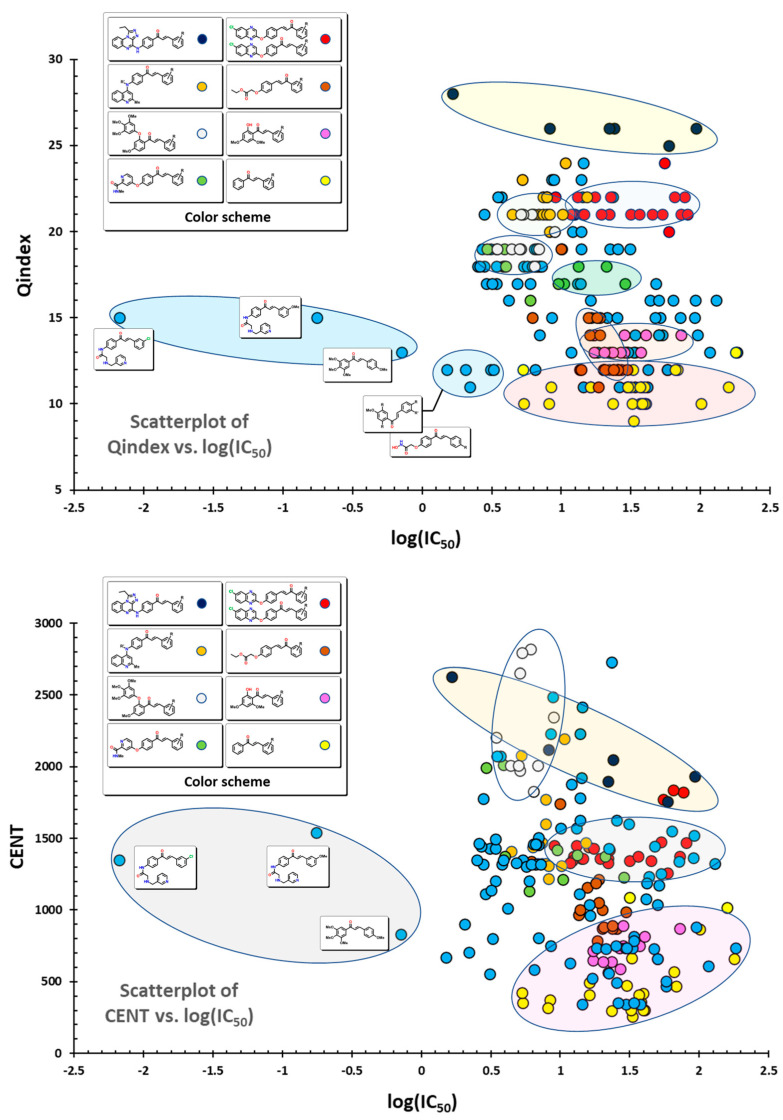
Scatterplots for the Qindex and the CENT descriptors against the logIC_50_ for the chalcone derivatives. Highlighted in colored dots are different sets of structurally similar compounds as from the color-scheme box. For both graphics, low-branching chalcones are seen at the bottom, while high-branching compounds are at the top.

The next two variables, Qindex and CENT, belong to the topological descriptors. Qindex is the quadratic index calculated by the normalization of the First Zagreb index (ZM1) [62]. On the other hand, Centralization (CENT) is defined as:

(2) $$CENT=2\left(W\right)-(nSK\times UNIP)$$

where W is the Wiener index, nSK the number of non-hydrogen atoms, and UNIP is the unipolarity of the molecule (defined as the minimum value of the vertex distance degrees) [63]. Both descriptors are mainly related to molecular branching. The scatterplot of the Qindex descriptor against the logIC_50_ is shown in Figure 5. The plot shows that chalcone derivatives with low branching are located at the bottom of the graph, while compounds with more complex functional groups (high branching) at the α-ring are seen at the middle top. A similar trend is observed for the CENT descriptor against the observed bioactivity, although the dispersion of the data is more pronounced. Nonetheless, the chemical space can be divided into three regions, where simple chalcone derivatives are at the bottom. Notably, for both plots, three compounds are seen at the middle-left of the scatterplot, near the low-branching chalcones. These chemical species have high potency against the MCF-7 cancer line, although they show complex functional groups seen farther from the rest of the high-branching compounds. For each scatterplot, chemical species which are structurally similar are highlighted in colored circles. For more structurally diverse chalcones, light-blue dots are dispersed across the graphic.

**Figure 6:**
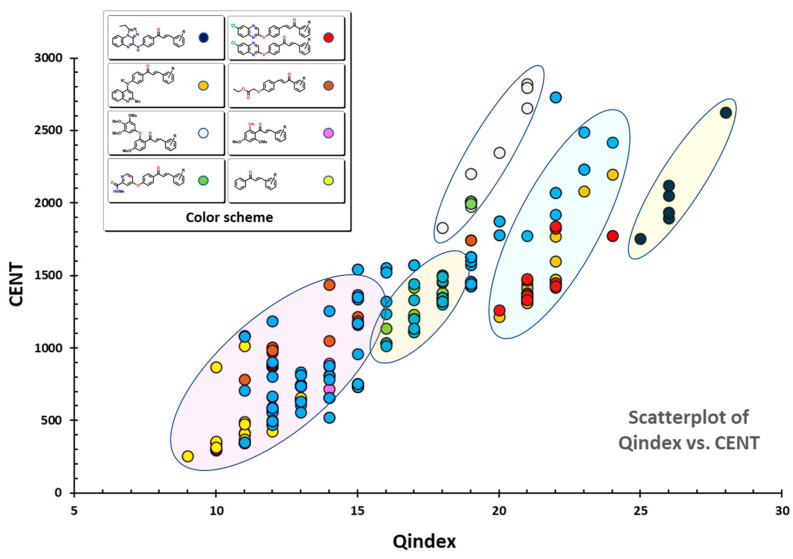
Scatterplot for the Qindex against CENT descriptors for the chalcone derivatives. Highlighted in circles are different sets of structurally similar compounds. Low-branched chalcones are seen at the left bottom while high-branching compounds are seen more dispersed at the top-right of the graphic. Structurally more diverse chalcones are depicted as light-blue dots, while similar sets of chalcones are colored circles.

From Figure 6, a correlation is seen between the CENT and Qindex. In this case, simple chalcones are located at the left-bottom of the graphic while gradually increasing in values, with the more complex chalcones at the top-right of the scatterplot.

Finally, two atom-centered fragments’ descriptors (ACF) are shown in Equation (1), C029 and H052. The C029 depicts a central sp^2^–carbon atom that is bonded to two electronegative atoms and one R group, with delocalized electron density between one X atom (X = N, O) and the R group. On the other hand, H052 is defined as a hydrogen atom bonded to a sp^3^-C atom next to a carbon atom bonded to an electronegative atom X.

**Figure 7:**
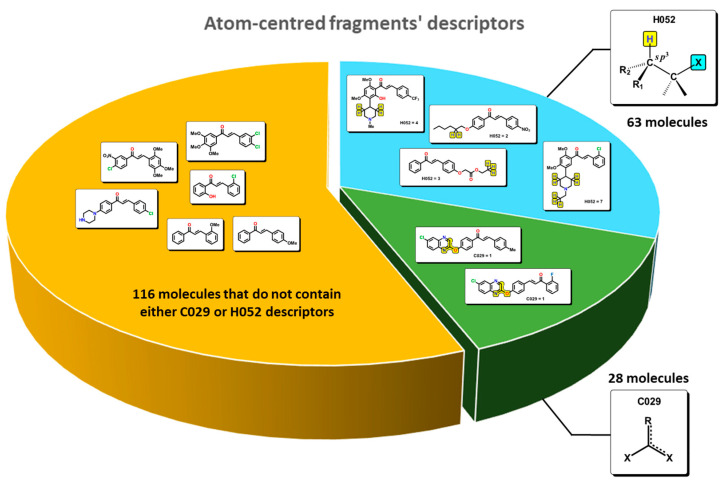
Atom-centered fragments (ACF) descriptors with representative molecules incorporate them within their structures. In light blue, 63 molecules (30.4%) have the H052 descriptor, while in green, 28 molecules (13.5%) of the 6-chloroquinoxalin-2-ol family are displayed. In yellow, 56% of the molecules (116 compounds) do not have any ACF descriptors.

Figure 7 shows the distribution of molecules that contain the C029 and the H052 descriptors. It is seen that 63 molecules from the original dataset have the H052 fragment (shown in light blue). On the other hand, only 28 molecules display the C029 descriptor, which belongs to a specific family of chalcones that have the 6-chloroquinoxalin-2-ol fragment (green slice). Finally, the rest of the chalcone derivatives, 116 molecules, do not contain any ACF descriptors. It is noteworthy to observe that complex molecules, with large functional groups or very branched molecules, possess one of the ACF descriptors, while small chalcones (low-branched) do not have any.

Until now, our QSAR model has shown the importance of the molecular shape and branching of chalcones, which inspired us to evaluate if another class of molecular descriptors (geometrical) may fit better in our QSAR model; this decision was based on the rigid and aromatic-like character of most of the chalcones. From this, Equation (3) was obtained, which includes four geometrical descriptors, and added the new mathematical model, which is shown below:

logIC_50_ = (0.4682 ± 0.0018)**R6_OH** + (−0.2255 ± 0.0002)**R2_OMe** + (0.3459 ± 0.0047)**R4_FA029** + (−0.7542 ± 0.0048)**R2_TMPhO** + (−0.0766 ± 0.0003)**R2′_Cl** + (−0.2831 ± 0.0002)**R4′_Cl** + (−0.1747 ± 0.0007)**R4′_FB035** + (−0.1301 ± 0.0001)**Qindex** + (0.0007 ± 0.0000)**CENT** + (0.2352 ± 0.0031)**C029** + (−0.0611 ± 0.0001)**H052** + (−0.1306 ± 0.0028)**J3D** + (−0.0139 ± 0.0009)**RGyr** + (0.7619 ± 0.0139)**SPH** + (−3.1858 ± 0.2521)**AROM** + 5.4341 ± 0.1496 (3)

$$n=152;\;R^{2}=85.554\;(\pm 0.3254);\;{R^{2}}_{\mathrm{ADJ}}=83.514\;(\pm 0.4245);\;a(R^{2})=0.0938\;(\pm 0.0000);\;\;s=0.155\;(\pm 0.0000);\;F=41.916\;(\pm 3.6835);\;{Q^{2}}_{\mathrm{LMO}}=80.534\;(\pm 0.4927);\;\;{Q^{2}}_{\mathrm{BOOT}}=78.186\;(\pm 0.6018);\;{Q^{2}}_{\mathrm{EXT}}=79.41\;(\pm 10.6679);\;a(Q^{2})=-0.2082\;(\pm 0.000);\;\;\delta K=0.0146\;(0.000);\;\delta Q=-0.0034\;(-0.005);\;R^{P}=0.06\;(0.100);\;R^{N}=-0.05\;(-0.056)$$

**Figure 8:**
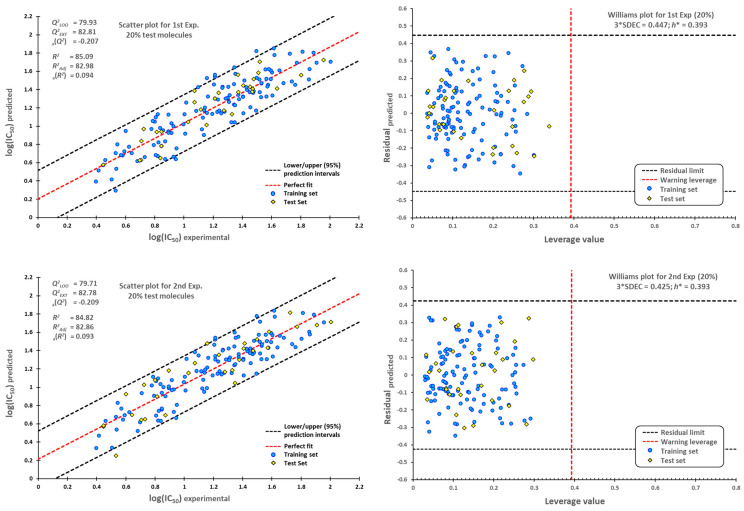
Selected scatterplots of the predicted logIC_50_ against experimental logIC_50_ values with their corresponding Williams plots for chalcone derivatives. Blue dots represent molecules of the training set (70%), and yellow diamonds depict molecules used for the test set (30%). The dotted vertical line in red indicates the warning leverage limit. The upper/lower dotted horizontal lines in black represent the boundaries for which the triple of the standard deviation (3*SDEC) value is used.

As from the previous QSAR, all statistical parameters were obtained as their average values, and all the parameters were satisfied; for this model, higher values of the statistical parameters were achieved. In Figure 8, selected scatterplots for the predicted logIC_50_ against experimental logIC_50_ values and their corresponding Williams plot are shown. These graphics show that this new QSAR model possesses a better predictive ability and a more extended applicability domain. For this, many of the chalcones that started in this study were removed due to their structural differences, according to the geometrical descriptors: AROM, J3D, SPH, and RGyr.

**Figure 9:**
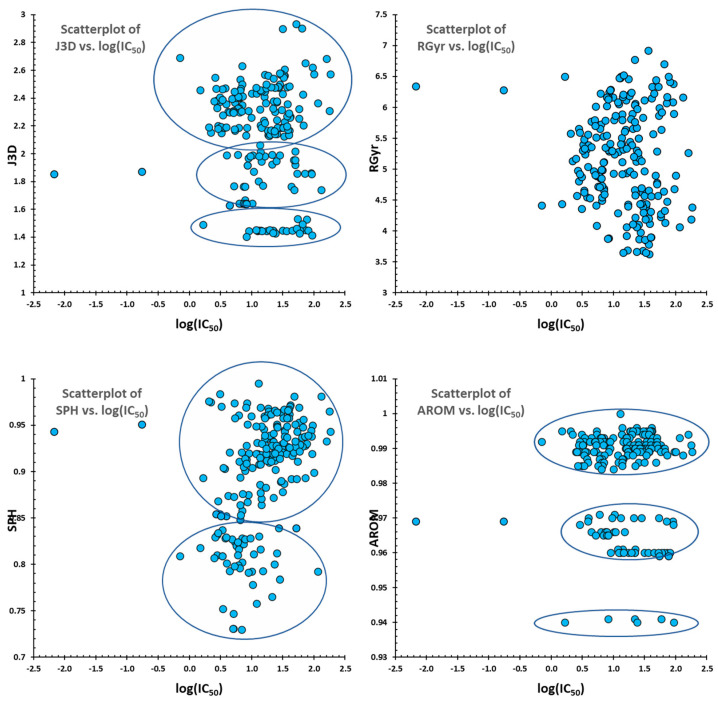
Scatterplots for the J3D, RGyr, SPH, and AROM descriptors against the logIC_50_ for the chalcone derivatives.

The 3D Balaban index (J3D) is obtained by the Balaban distance-connectivity index *J* (Equation (4)) using the geometric distance degrees (d_x_) in place of topological distance degrees.

(4) $$\;J=\frac{M}{\mu +1}\sum_{ij}{\left(d_{i}d_{j}\right)}^{-\frac{1}{2}}$$

where *M* is the number of edges, and *µ* is the cyclomatic number (number of rings) [64]. *J* is a graph invariant. This descriptor is highly discriminant, and its value does not change substantially with molecule size or number of rings. According to our QSAR model, molecules with higher J3D values are expected, and from Figure 9, the J3D range values and examples of higher J3D values from molecules are displayed.

The radius of gyration (RGyr) is a size descriptor for the distribution of atomic masses in a compound; its calculation is as follows:

(5) $$RGyr=\sqrt{\frac{\sum_{I=1}^{nAT}m_{i}\cdot r_{i}^{2}}{MW}}$$

where $r_{i}^{2}$ is the distance of the *i*th atom from the center of mass, $m_{i}$ is the corresponding atomic mass, nAT the atom number, and *MW* the molecular weight [65]. In our QSAR model, this descriptor is positively related to the potency of the chalcones, this is, increasing the RGyr, the biological activity of the chalcone will increase. In Figure 9, the RGyr range value and some of the chalcone structures are displayed.

The spherosity (SPH) is an anisometry descriptor calculated by the eigenvalues of the covariance matrix calculated from the molecular matrix:

(6) $$SPH=\frac{3\lambda_{3}}{\lambda_{1}+\lambda_{2}+\lambda_{3}}$$

The spherosity index goes from zero for flat compounds (benzene) to one for totally spherical molecules. SPH has a positive coefficient meaning that as molecules tend to adopt a spherical form, the expected biological activity decreases (as the logIC_50_ increases). [66]. This characteristic is related to our previous QSAR analysis, where more rigid and cylinder-like structures are preferred (vide supra).

The Aromaticity (AROM) is derived from the general aromaticity indices:

(7) $$AROM=1-\frac{100/35}{{\bar{r}}^{\pi }}\cdot \sqrt{\frac{\sum {{(r}^{\pi }-{\bar{r}}^{\pi })}^{2}}{B_{\pi }}}$$

where the sum runs over the bonds belonging to aromatic rings, ${\bar{r}}^{\pi }$ is the π bond average length and $r^{\pi }$ are the actual π bond lengths, and $B_{\pi }$ is the number of aromatic bonds. In the chalcone derivatives, the AROM descriptor possesses a negative coefficient indicating that at great aromaticity values, the better the molecule’s activity. For most derivatives, the chalcone core is planar; hence, a better overlap of π orbitals is expected [67].

An in-depth analysis of the four new descriptors versus their logIC_50_ showed that structures are arranged such that low-branching molecules are located at the top of their corresponding plots, while complex functional groups are at the bottom (Figure 9). It is especially seen within the J3D, SPH, and AROM descriptors, while for RGyr, more dispersion of the data is obtained. When J3D and AROM descriptors are plotted against each other, three regions are observed, where specific limits are seen for each of the *x*, *y*-axis: from left to right, 1.444 and 2.901 are the limits for the J3D Balaban index (*x*-axis), while below 0.959 and above 0.996 are molecules with the AROM descriptor (*y*-axis). Chalcone derivatives of the tetraazatricyclo- and the *ortho*-aminophenyl-motifs are seen outside these limits, which are in agreement with the outliers observed from Williams’ plots.

**Figure 10:**
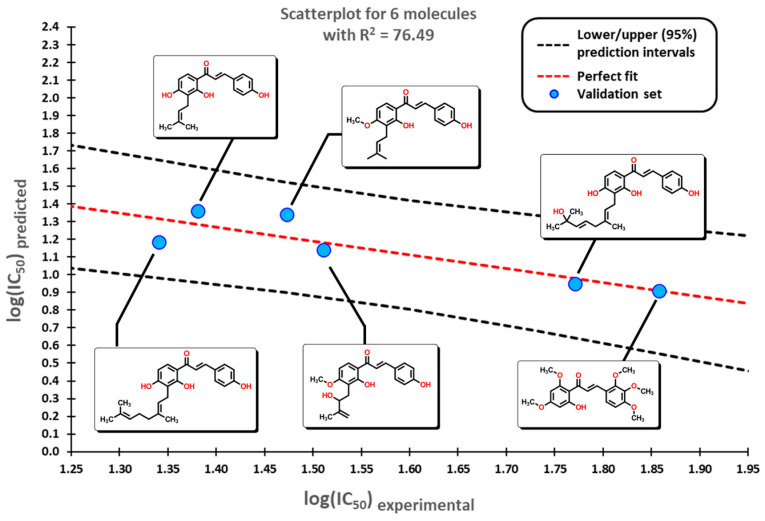
Scatterplot for the molecular dataset of some chalcone derivatives. Selected molecules are displayed within the plot showing the change in substituents.

In order to test the predictive ability of the QSAR model, a set of molecules not introduced in the initial dataset of compounds was used as an external validation: compounds reported by Sangpheak [68], Xiao [69], and Kumar [70]. As seen in Figure 10, there is a good correlation between the predicted values and the corresponding experimental data (Table S2), with a $R^{2}$ value of 75.62, allowing us to confirm the reliability of our model. This strategy has been used in other work to ensure its predictive ability with positive results [71].

### 2.3. Design and Synthesis of 2′-Hydroxy-4′-cinnamate Chalcone

According to the QSAR model of Equation (3), we believe that new hybrid molecules containing 2′,4′-DHC and polyphenol derivatives may be a good choice in the search for cytotoxic agents. In this study, we synthesized the parent 2′,4′-dihydroxychalcone (**A**) and the hybrid cinnamate chalcone **B**. The modification was carried out in the C-4’ of ring α, leaving intact ring β of the chalcone. We chose cinnamic acid because of its reported antioxidant activity. The decision to only modify position 4 of the 2′,4′-DHC was due to the fact that in our previous work, we found that modification of the chalcone at this position improved the cytotoxic activity against PC-3 (prostate) cancer cell line [20].

**Scheme 1:**
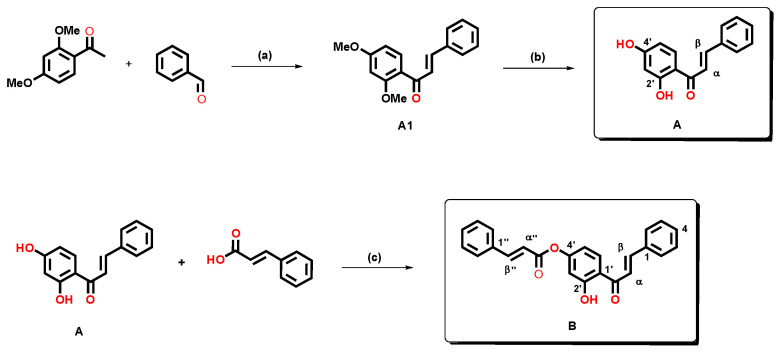
Synthesis of 2′,4′-dihydroxychalcone (**A**) and 2′-hydroxy-4′-cinnamate chalcone (**B**). Reagents and conditions: (a) KOH, MeOH, reflux 4 h; (b) BBr_3_, CH_2_Cl_2_ (anhydrous), −78 °C for 10 min, then at r.t. for 24 h. (c) DCC, DMAP, CH_2_Cl_2_/THF (anhydrous), rt, 2 h, N_2_.

The target 2′-hydroxy-4′-cinnamate chalcone hybrid (**B**) was synthesized in two steps (Scheme 1). Firstly, 2′, 4′-dihydroxy chalcone (**A**) was synthesized via base-catalyzed Claisen–Schmidt condensation of 2,4-dimethoxy-acetophenone with benzaldehyde, followed by demethylation with BBr_3_ in anhydrous CH_2_Cl_2_, as described [20]. The 4′-cinnamoyl-2′-hydoxy-chalcone (**B**) was obtained by an esterification reaction using cinnamoyl chloride and 2′,4′-DHC (**A**) in the presence of DCC and DMAP. Chalcones **A** and **B** were characterized by 1D and 2D NMR and mass spectra analysis.

### 2.4. Biological Activity

The synthesized chalcones **A** and **B**, as well as the protected derivative **A1** with purity values over 95%, were evaluated by the MTS assay for their capacity to inhibit the in vitro growth of breast (MCF-7), lung (A-549), cervical (CaSki), and liver (Hep 3B, HepG2) human cancer cell lines. We also included immortalized human hepatocyte cell line (IHH) as a control of non-cancerous cells. Experimental results and predicted activity are summarized in Table 1, indicating that chalcones display a better effect against HepG2, Hep3B (liver), and MCF-7 (breast) cell lines. Chalcone 2′,4′-DHC (**A**) showed better activity against liver (HepG2 and Hep3B) and cervical (CasKi) cell lines with IC_50_ values of 58.33, 54.16, and 58.33 µM, respectively. In addition, compound **B** was more potent than the parent compound **A** on all tested cell lines. Indeed, chalcone **B** is more potent in the liver (HepG2 and Hep3B) and breast (MCF-7) cell lines with IC_50_ values of 11.89, 10.27, and 14.86 µM, respectively. Comparing the results obtained experimentally for **A** and **B** in the MCF-7 cell line with those obtained in the QSAR model in Equation (3), we can see a difference of ca. 7 and 3 units, respectively. Thus, QSAR was able to qualitatively predict the best of the four proposed derivatives, and this is corroborated by the results obtained in the MCF-7 cell line of derivative **B**. Both compounds present less activity on the lung cancer cell line, with IC_50_ values of 112.5 µM for compound **A** and 56.75 µM for compound **B.**

**Figure 11:**
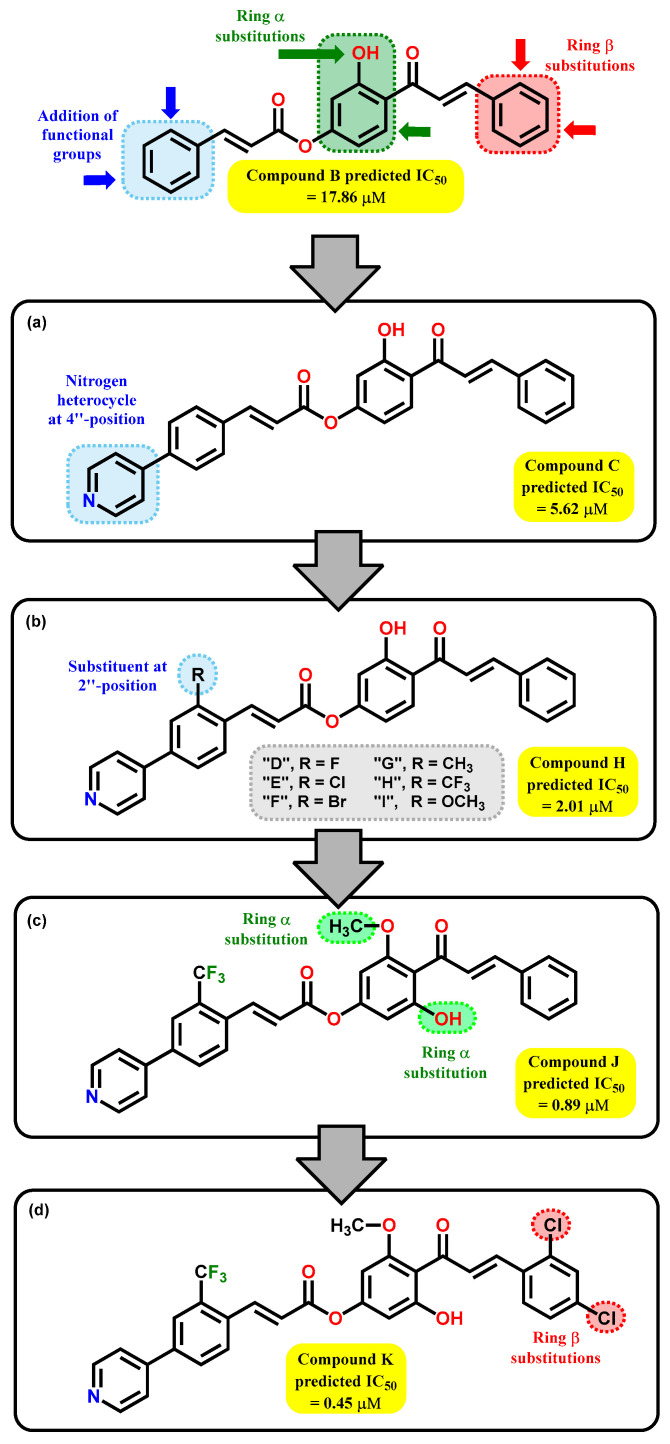
Proposed chalcone-cinnamate derivates (**B**–**K**), which, in accordance with the QSAR model, are expected to be high in potency compared to parent compound **B**. In (**a**,**b**), the introduction of pyridine and trifluoromethyl groups decreases the IC_50_ of the compound as the values of Qindex and CENT increase, respectively; (**c**) the methoxy and hydroxyl functional groups at positions 2 and 6 on the α-ring of the chalcone core enhances potency in compound **J**; finally, in (**d**) chlorine atoms on β-ring are beneficial for the expected IC_50_ value.

Because of the high potency of compound **B**, we decided to explore structural modifications on the chalcone derivative using specific functional groups according to the QSAR model described above (Equation (3)). Simple chemical substitutions can be performed on the aromatic ring of the cinnamoyl motif. First, because a high number of approved drugs contain nitrogen heterocycles, which can induce hydrogen bonds with the receptor, we decided to evaluate pyridine rings as a starting choice, with the replacement of the *p*-hydrogen atom at the cinnamoyl ring (Figure 11a, compound **C**). Its predicted IC_50_ value decreases from 17.86 to 5.62 µM. As a second approach, different groups were evaluated to determine their potency compared to the parent compound. It is estimated that the trifluoromethyl group can enhance the potency of the **B**-derivative, decreasing the expected value to 2.01 µM (Figure 11b, compound **H**). According to Equation (1), the presence of methoxy and hydroxyl functional groups at positions 2 and 6 on the α-ring of the chalcone core enhances the potency of the compounds by decreasing the expected IC_50_ value as seen in compound **J** (Figure 11c). This also corresponds to an increase in the Qindex and CENT values, which agree to a larger compound branching. Finally, the QSAR model points out two chlorine atoms on the β-ring, which can be beneficial to the predicted cytotoxic value; this is observed for the expected value, which decreases to 0.45 µM in compound **K** (Figure 11d). Calculated molecular descriptors and predicted logIC_50_ values are shown in Table S3.

### 2.5. In Silico ADME/tox Studies of A–K

ADME/tox data (absorption, distribution, metabolism, excretion, and toxicity) can provide information on how the human body treats a drug. In silico pharmacokinetic parameters can predict drug candidates and drug optimization [72]. We decided to analyze ADME/tox data of the synthetic chalcones **A** and **B** and the proposed chalcone-cinnamate derivatives according to the QSAR study (**C**–**K**) (Table 2). Drug-likeness analysis showed that only the synthetized chalcones **A** and **B** have not violated Lipinski, Veber and Ghose, suggesting that they would display well-behaved absorption or permeation [73]. In contrast, **F**, **H**, **J,** and **K** have one Lipinski violation (exceeded in MW). Lipophilicity was assessed using the logarithm of the n-octanol/water partition coefficient (LogPoctanol/water). Chalcones presented an MLogP range from 2.17 to 3.79 and are related to high membrane permeability. On the other hand, only chalcones **A** and **B** comply with Veber rules, with a rotatable bond of less than 10, which indicates molecular flexibility to their target. Moreover, a TPSA range between 57.53 and 85.72 Å (TPSA < 140 Å) [73] means high oral bioavailability for all compounds.

Moreover, most of them (except for **F**, **H**, **J,** and **K**) could have a high gastrointestinal absorption. The presence of the BBB (blood–brain barrier) makes it challenging to develop new treatments for brain diseases, including radiopharmaceuticals for the brain; according to our results, only **A** and **B** could cross BBB [74]. P-glycoprotein (P-gp) is a multi-specific efflux transporter and can modulate the pharmacokinetics of anti-cancer drugs. It is related to multidrug resistance in human multidrug-resistant (MDR) cancers [75]. Only chalcone **H**, **J,** and **K** could act as P-gp substrates.

Finally, all designed chalcones (except **D**, **F,** and **K**) do not present hepatotoxicity (liver damage). All of them have non-mutagenicity or carcinogenicity, presenting an LD_50pred_ from 1000 mg/kg to 3800 mg/kg. In general, according to ADME predictions, the synthesized chalcones **A** and **B** are the best candidates and could have the required cell membrane permeability and bioavailability without toxicity, followed by **C** and **G**.

## 3. Material and Methods

### 3.1. Calculation of the Molecular Descriptors

The structures of the molecules of interest were drawn in Avogadro and MarvinSketch (ChemAxon, Budapest, Hungary). The molecules were analyzed in the program Dragon 05 [76], where the families of molecular descriptors corresponding to constitutional properties, topological, functional groups, geometric, atom-centered fragments, molecular charge, and molecular properties were obtained (Table 3). Additionally, for rings α and β, indicative variables were used as descriptors: as stated above, if a specific functional group appeared at a particular position in the ring, a number 1 was assigned; otherwise, a 0 value was designated.

### 3.2. DataSet

An initial dataset of 207 compounds was obtained from the literature between 1995 and 2022; information can be seen in the Supplementary Materials. These compounds shared the same evaluation method against cancer line MCF-7. To improve the reliability of the data, the compounds were curated to eliminate outliers, uncertainties, and potential errors. Data used for the model generation required IC_50_ values in μM; if μg/mL data were reported, transformation into their corresponding μM was done, and finally all were transformed to logIC_50_, leaving a final set of 160 molecules for the first model and 152 for the second model.

### 3.3. Construction of QSAR Model

The QSAR model was constructed using the genetic algorithms (GA) technique in MobyDigs program [61]. All molecular descriptors were used as independent variables (*x*), and the experimental cytotoxic activity was the dependent variable (*y*). The selection of the best model was based on parameter values such as the coefficient of determination ($R^{2}$), the standard deviation (s), and the Fischer test (F). The Y-scrambling test was used to guarantee that the QSAR model was built adequately in terms of correlation obtained by chance. This was performed firstly by randomly permuting the logIC_50_ values of the dataset and then using the new column of values with the same variables to generate new models. The procedure was repeated 300 times, and the quality parameters of these new models were compared to the original values of the QSAR model: if the original model has no chance correlation, the new $R^{2}$ and $Q^{2}$ values calculated for the permuted logIC_50_ QSAR models will have a significant difference with respect to the original values; otherwise, the model is rejected. Non-collinearity between descriptors is determined using the QUIK rule. The typical δK threshold values for models are between 0.01–0.05. Models that have negative values are not allowed. To detect models with an excess of “good” or “bad” descriptors, the redundancy ($R^{P}$) and overfitting (***R^N^***) rules were applied.

### 3.4. Statistical Validation

The coefficient of determination, $R^{2}$. The square of the correlation coefficient (also called Pearson’s *r*) between the observed and predicted values in regression is calculated as shown in Equation (8) [57]. Acceptable values of $R^{2}$ should be $R^{2}\geq 0.6$ [77,78].

(8) $$R^{2}=1-\frac{\sum_{i-1}^{n}{\left({\hat{y}}_{i}-y_{i}\right)}^{2}}{\sum_{i-1}^{n}{\left(y_{i}-\underline{y}\right)}^{2}}$$

Fisher function *F*. Among most statistical tests, it is defined as the ratio between the model sum of squares *MSS* and the residual sum of squares *RSS*:

(9) $$F=\frac{\sum_{i=1}^{n}\frac{{\left({\hat{y}}_{i}-\underline{y}\right)}^{2}}{d\int_{M}}}{\sum_{i=1}^{n}\frac{{\left({\hat{y}}_{i}-y_{i}\right)}^{2}}{d\int_{E}}}=\frac{\frac{MSS}{d\int_{M}}}{\frac{RSS}{d\int_{E}}}$$

where $d\int_{M}$ and $d\int_{E}$ refer to the degrees of freedom of the model (molecular descriptors in the model) and error, respectively, while $\underline{y}$ is the average value of experimental activity. The calculated value is compared with the critical value *F*_crit_ for the corresponding degrees of freedom. It is a comparison between the model-explained variance and the residual variance: high values of the F test indicate reliable models [79].

The standard deviation “s” is a measure of the dispersion, or scatter, of the data used to determine the variation in values that respect a mean value, as shown in Equation (10) [55].

(10) $$s=\frac{\sum_{i=1}^{n}{\left({\hat{y}}_{i}-y_{i}\right)}^{2}}{n-2}$$

where variable n represents the number of molecules in the study, ${\hat{y}}_{i}$ represents the experimental activity $y_{i}$ is the calculated activity from the QSAR model.

In addition, for the validation of our statistical model was required the analysis of the rules of QUIK, redundancy, and overfitting. The QUIK rule is based on the *K* multivariate correlation index that measures the total correlation of a set of variables [59] defined as:

(11) $$K=\frac{\sum_{j}\left[\frac{\lambda_{j}}{\sum_{j}\lambda_{j}}-\frac{1}{p}\right]}{\frac{2\left(p-1\right)}{p}}\;\;\;\;j=1,\ldots ,p\;\;\;\;and\;\;\;0\leq K\leq 1$$

where λ are the eigenvalues obtained from the correlation matrix of dataset X (*n*, *p*), being *n* the number of objects and *p* the number of variables. The total correlation in the set given by the model predictors X plus the response Y (*K*_XY_) should always be greater than that measured only in the set of predictors (*K*_X_). Therefore, if $K_{XY}-K_{X}<\delta K$ the model is rejected, where δK has values of 0.01 to 0.05; models with negative differences are unacceptable [58].

The redundancy rule ($R^{P}$) detect models with an excess of “good” predictors as well and establishes that if $R^{P}<t^{P}$, the model is rejected. Depending on the data, the $t^{P}$ values range from 0.01 to 0.1. $R^{P}$ is defined by:

(12) $$R^{P}=\prod_{j=1}^{p^{+}}\left(1-M_{j}\left(\frac{p}{p-1}\right)\right)\;\;\;\;\;\;\;\;\;Mj>0\;\mathrm{and}\;0\leq R^{N\;}\leq 1$$

The overfitting rule (***R^N^***) tells us that if $R^{N}<t^{N}\left(\varepsilon \right)$ the model is rejected, then the $t^{N}$(ε) values are calculated by Equation (13).

(13) $${\;t}^{N}\left(\varepsilon \right)=\frac{p\varepsilon -R}{pR}$$

where *ε* values range from 0.01 to 0.1, and *p* is the number of variables in the model $R^{N}$ is defined by:

(14) $$R^{N}=\sum_{j=1}^{p^{-}}M_{j}\;\;\;\;\;\;\;\;\;Mj<0\;\mathrm{and}-1<R^{N}\leq 0\;$$

where $M_{j}$ is defined by Equation (15):

(15) $$KM_{j}=\frac{R_{jy}}{R}-\frac{1}{P}\;\;\;and\;-\frac{1}{P}\leq M_{j}\leq \frac{p-1}{p}$$

$R_{jY}$ is the absolute value of the regression coefficient between the *j*th descriptors and the response Y. If a descriptor has a high correlation with the response, $R^{P}$ takes the low value and is close to 0 when $R_{jY}$ is equal to R and p>1. $R^{N}$ accounts for an excess of noisy or useless variables and can be thought of as a measure of overfitting. It takes the maximum value to zero when non-noisy variables are in the model [60].

### 3.5. External Validation

The generated model was validated externally by the prediction of a set of molecules that were not included in the generation of the model. As stated above, data curation was performed on a total of 10 molecules drawn in Avogadro, and their molecular descriptors were obtained from the Dragon software package, as well as an analysis using the Free-Wilson approach. A complete list of descriptors and references can be found in Supplementary Materials Table S1.

### 3.6. Chemistry

All commercial regents: 2,4-dihydroxyacetophenone (99%), benzaldehyde (99%), cinnamic acid, benzyl bromide, dicyclohexylcarbodiimide (99%) dimethyl sulfoxide, potassium carbonate, potassium hydroxide, dichloromethane anhydrous (99%), methanol and boron tribromide were obtained from Sigma–Aldrich (St. Louis, MO, USA) and were used without further purification. Melting points were determined in a Prendo and were uncorrected. Nuclear magnetic resonance spectra were determined at 600 MHz for ^1^H NMR and 150 MHz for ^13^C NMR in the presence of tetramethylsilane (TMS) as an internal standard in CDCl_3_ on a Bruker AMX 600 instrument. Chemical shifts δ are expressed in parts per million (ppm) relative to TMS and coupling constants (J) in Hertz. Multiplicities are indicated as singlet (s), doublet (d), triplet (t), quartet (q), double of double (dd), multiplet (m), and broad singlet (bs). Open column chromatographies were carried out on silica gel 60 (70–230 mesh), and different solvent systems of *n*-hexane and EtOAc were used as mobile phases for the purification. Mass spectra were obtained in a Jeol M-station JEOL JMX-AX 505 HA mass spectrometer and HPLC Agilent Infinity 1260 coupled Quadrupole LC/MS 6120.

#### 3.6.1. Synthesis of 2′,4′-Dihydroxychalcone (**A**)

The 2,4-Dimethoxy acetophenone was obtained by alkylation reaction of acetophenone (1.0 g, 6.58 mmol) with methyl iodide (0.8 mL, 13.0 mmol), using K_2_CO_3_ (4.54 g, 32.8 mmol) as a base, in MeOH (10 mL). The reaction mixture was stirred at 25 °C for 24 h. The crude reaction was purified by column chromatography on silica gel eluting with 99:1 to 98:2 (*n*-hexane-EtOAc) to yield 23.3 mg (11.6%) of 2,4-dimethoxy acetophenone. Chalcone **A** was prepared by Claisen–Schmidt condensation of benzaldehyde (0.8 mL, 7.8 mmol) and 2,4-dimethoxyacetophenone (2.0 g, 6.02 mmol), in the presence of KOH (1.0 g, 18.055 mmol) in MeOH (15 mL) at reflux for 4 h. The reactions were monitored by TLC and purified by column chromatography 100:0 to 97: 3 (*n*-hexane-EtOAc) to give 2′,4′-dimethoxy chalcone (**A1**) as yellow solid (1.92 g, 84%). Then, a deprotection reaction was performed using dimethoxy chalcone (0.06 g, 0.22 mmol), 1M solution of BBr_3_ (1.68 mL, 1.68 mmol), and anhydrous CH_2_Cl_2_ (5 mL), the reaction was stirred at −78 °C for 10 min and at room temperature for 48 h, monitoring by TLC and purified by column chromatography 100:0 to 97:3 (n-hexane-EtOAc) to afford A as yellow solid (0.0133 g, 25% yield). NMR data were in accordance with those described in the literature [20].

#### 3.6.2. Synthesis of 2′-Hydroxy-4′-cinnamate Chalcone (**B**)

A solution of cinnamic acid (0.0082 g, 0.055 mmol), dicyclohexylcarbodiimide (0.017 g, 0.083 mmol), DMAP (0.0067 g, 0.055 mmol), and anhydrous CH_2_Cl_2_ (5 mL) was stirred at rt by 30 min. Subsequently, it was dropped into the reaction flask 2′,4′-DHC (**A**) (0.0133 g, 0.055 mmol) in THF (2 mL) and left in stirring for 2 h; it was monitoring by TLC and purified by column chromatography 100:0 to 98:2 (*n*-hexane-EtOAc) to afford **B** as yellow solid (0.0038 g, 17. 24%). ESI+MS, *m*/*z*: [M]+1 (371).

2″-Hydroxy-4′-cinnamate chalcone (**B**). Yellow solid (0.0038 g, 17. 24%). m.p. 130–132 °C. ^1^H NMR (600 MHz, CDCl_3_) δ: 13.08 (s, OH-2′), 7.96 (1H, d, *J* = 9.0 Hz, H-6′), 7.94 (1H, d, *J* = 15.4 Hz, H-β), 7.88 (1H, d, *J* = 16.0 Hz, H-β″), 7.69-7.64 (4H, m, H-3, H-5, H-3″, H-5″), 7.61–7.57 (2H, m, H-4, H-4″), 7.61 (1H, d, *J* = 15.8 Hz, H-α), 7.46–7.42 (4H, m, H-2, H-6, H-2″, H-6″), 6.87 (1H, d, *J* = 8.7, 2.3 Hz, H-3′), 6.81 (1H, dd, *J* = 8.7, 2.3 Hz, H-5′), 6.61 (1H, d, *J* = 16.0 Hz, H-α″). ^13^C NMR (150 MHz, CDCl_3_) δ: 192.9 (C-7), 165.32 (C-2′), 164.54 (C-7″), 156.9 (C-4′), 147.58 (C-α″), 145.74 (C-α), 134.61 (C-1), 134.05 (C-1″), 131.12 (C-6′), 131.08 (C-2, C-6), 130.99 (C-2″, C-6″), 129.17 (C-3, C-5), 129.16 (C-3″, C-5″)128.81 (C-4), 128.51 (C-4″), 120.11 (C-α), 118.03 (C-1′), 116.91 (C-α″), 112.17 (C-5′), 111.44 (C-3′). ESI+MS, *m*/*z*: [M]+1 (371). Anal. calcd. for C_24_H_18_O_4_: C, 77.82; H, 4.90. Found: C, 77.80; H, 5.03.

### 3.7. Antiproliferative Activity

Compounds **A** and **B**, as the first approach compounds, were subjected to antiproliferative trials in cell lines of cancer such as Hep3b, HepG2 (hepatocellular carcinoma), MCF-7 (breast), A549 (lung), and Caski (cervical), human cancer cell lines, obtained from ATCC (American Type Culture Collection, Manassas, VA, USA). We also included an immortalized human hepatocytes cell line (IHH) as a control of non-cancerous cells [80]. CaSKi cells were grown in RPMI-1640 medium (Sigma–Aldrich, St. Louis, MO, USA), A549 cells Kaighn’s Modification of Ham’s F-12 Medium (American Type Culture Collection, Manassas, VA, USA), while Hep3B, HepG2, IHH, and HeLa in a DMEM medium (Invitrogen, Thermo Fisher Scientific, Inc., Waltham, MA, USA) and supplemented with fetal bovine serum 10% (SFB, Invitrogen) and with 2 mM glutamine, all cultures were incubated at 37 °C in an atmosphere of 5% CO_2_.

They were cultivated in plates of 96 wells, 5000 cells per well, to initiate the evaluation. The concentrations used for the compounds were 100, 10, 1, 0.1, and 0.001 μg/mL for a dose/response curve and were incubated at 37 °C in 5% of CO_2_ atmosphere for 48 h. The number of viable cells in the proliferation was determined using the Kit CellTiter 96^®^ Aqueous One Solution Cell Proliferation Assay (Promega) in accordance with the instructions of the manufacturer. Then, the absorbance values were measured at 490 nm in an automatic microplate ELISA reader (Promega, Madison, WI, USA). Experiments were conducted in triplicate in three independent experiments. The data were analyzed in the statistical program Prisma 6.01, and the mean inhibitory concentration (IC_50_) was determined by regression analysis.

### 3.8. In Silico ADME/tox

The freely accessible SwissADME web tool [81] (http://www.swissadme.ch/ (accessed on 20 April 2018)) was used for ADME data, and Protox II [82] (https://tox-new.charite.de/protox_II/ (accessed on 20 April 2018)) was used for toxicology parameters. Chemical structures of compounds **A**–**K** were introduced into the SwissADME web to obtain the SMILE nomenclature. We selected the most important ADME/Tox properties to determine lipophilicity, pharmacokinetics, and toxicology.

## 4. Conclusions

Our QSAR model showed a good predictive capability supported by the statistical rules. The mathematical model consists of fifteen molecular descriptors R6_OH, R2_OMe, R4_FA026, R4_FA029, R4_1PPD, R2_TMPhO, R2′_Cl, R4′_Cl, R2′_OMe, R4′_4MPPZ, R4′_FB035, Qindex, CENT, C029, and H052. The mathematical model shows how the presence or absence of different functional groups within the chalcone structure affects its cytotoxic activity against the MCF-7 cancer cell line. Aromaticity and molecular branching have a direct implication on the observed bioactivity. Our second QSAR model properly considered these properties of the chalcone and cinnamic acid cores by adding geometrical descriptors (J3D, RGyr, SPH, and AROM). QSAR model predicted IC_50_ values of 55.95 and 17.86 µM corresponding to the chalcones **A** and **B,** respectively, which were synthesized, obtaining yields of 34% and 24%, respectively. These compounds were evaluated under MTS assay on four lines of human cancer, liver (HepG2, Hep3B), breast (MCF-7), lung (A-549) and cervical (CasKi), and a non-cancerous line of the liver (IHH). The results of the biological test on compound **B** showed better activity in all cancer cell lines, as predicted by the QSAR model developed in this work. Finally, **B** was chosen as a molecular scaffold for the generation of new derivatives with enhanced predicted cytotoxic activity, with the addition of functional groups in accordance with the QSAR model (**C**–**K**), a drug-likeness analysis suggested that chalcones **A** and **B**, flowed by **C** and **G** are the best candidates to be synthesized.

## Figures and Tables

**Table 1:** Antiproliferative activity (IC_50_ values) of synthetic chalcones **A**, **A1**, and **B** against four human tumor cell lines and one no tumoral (IHH). All experimental and predicted values are expressed as µM.

Compound	IC_50_ (Mean ± S.D., µM)						IC_50_ Pred
	IHH	HepG2	Hep3B	Caski	A-549	MCF-7	MCF-7
A	100 ± 2.2	58.33 ± 2.5	54.16 ± 2	58.33 ± 3	112.5 ± 2.5	62.5 ± 4	55.95
A1	140.47 ± 4.5	109.52 ± 3.0	90.47 ± 4.0	114.28 ± 3.0	185.71 ± 7.0	88 ± 5.0	1.23
B	56.76 ± 2	11.89 ± 0.4	10.27 ± 0.2	29.72 ± 1.4	56.75 ± 2.3	14.86 ± 0.5	17.86
Paclitaxel *	0.08 ± 0.005	0.0065 ± 0.0007	0.008 ± 0.001	0.007 ± 0.0009	0.0352 ± 0.007	0.0395 ± 0.0046	-

* Paclitaxel positive control.

**Table 2:** In silico ADME/tox properties of **A** and **B** chalcones and proposed derivatives **C** to **K**.

Molecule	MLOGP ^a^	TPSA ^b^	BBB ^c^	Pgp Substrate ^d^	GI Absorption ^e^	Lipinski Violations	Ghose Violations	Veber Violations	Mutagenicity	Carcinogenicity	Hepatotoxicity
**A**	2.17	57.53	Yes	No	High	0	0	0	Inactive	Inactive	Inactive
**B**	3.22	63.6	Yes	No	High	0	0	0	Inactive	Inactive	Inactive
**C**	2.79	76.49	No	No	High	0	2	0	Inactive	Inactive	Inactive
**D**	3.15	76.49	No	No	High	0	2	0	Inactive	Inactive	Active
**E**	3.24	76.49	No	No	High	0	3	0	Inactive	Inactive	Inactive
**F**	3.34	76.49	No	No	Low	1	3	0	Inactive	Inactive	Active
**G**	2.98	76.49	No	No	High	0	2	0	Inactive	Inactive	Inactive
**H**	3.52	76.49	No	Yes	Low	1	3	0	Inactive	Inactive	Inactive
**I**	2.43	85.72	No	No	High	0	3	0	Inactive	Inactive	Inactive
**J**	3.16	85.72	No	Yes	Low	1	3	1	Inactive	Inactive	Inactive
**K**	3.79	85.72	No	Yes	Low	1	3	1	Inactive	Inactive	Active

^a^ MLogP = logarithm of compound partition coefficient between n-octanol and water; ^b^ TPSA = topological polar surface area; ^c^ BBB = blood–brain barrier penetration; ^d^ Pgp = permeability glycoprotein; ^e^ GI absorption = gastrointestinal absorption.

**Table 3:** Molecular descriptors calculated with Dragon 05 program.

Molecular Descriptors	Descriptor Family	Program
ZM1, ZM1V, ZM2, ZM2V, Qindex, SNar, HNar, GNar, Xt, Dz, Ram, Pol, LPRS, VDA, MSD, SMTI, SMTIV, GMTI, GMTIV, Xu, SPI, W, WA, Har, Har2, QW, TI1, TI2, STN, HyDp, RHyDp, w, ww, Rww, D/D, Wap, WhetZ, Whetm, Whetv, Whete, Whetp, J, JhetZ, Jhetm, Jhetv, Jhete, Jhetp, MAXDN, MAXDP, DELS, TIE, S0K, S1K, S2K, S3K, PHI, BLI, PW2, PW3, PW4, PW5, PJI2, CSI, ECC, AECC, DECC, MDDD, UNIP, CENT, VAR, BAC, Lop, ICR, D/Dr03, D/Dr04, D/Dr05, D/Dr06, D/Dr07, D/Dr08, D/Dr09, D/Dr10, D/Dr11, D/Dr12, T(N..N), T(N..O), T(N..S), T(N..F), T(N..Cl), T(N..Br), T(N..I), T(O..O), T(O..S), T(O..F), T(O..Cl), T(O..Br), T(S..S), T(S..F), T(S..Cl), T(S..Br),T(F..F), T(F..Cl), T(F..Br), T(Cl..Cl), T(Cl..Br), T(Br..Br)	Topological	DRAGON
W3D, J3D, H3D, AGDD, DDI, ADDD, G1, G2, RGyr, SPAN, SPAM, MEcc, SPH, ASP, FDI, PJI3, L/Bw, SEig, HOMA, RCI, AROM, HOMT, DISPm, QXXm, QYYm, QZZm, DISPv, QXXv, QYYv, QZZv, DISPe, QXXe, QYYe, QZZe, DISPp, QXXp, QYYp, QZZp, G(N..N), G(N..O), G(N..S), G(N..P), G(N..F), G(N..Cl), G(N..Br), G(N..I), G(O..O), G(O..S), G(O..P), G(O..F), G(O..Cl), G(O..Br), G(O..I), G(S..S), G(S..P), G(S..F), G(S..Cl), G(S..Br), G(S..I), G(P..P), G(P..F), G(P..Cl), G(P..Br), G(P..I), G(F..F), G(F..Cl), G(F..Br), G(F..I), G(Cl..Cl), G(Cl..Br), G(Cl..I), G(Br..Br), G(Br..I), G(I..I)	Geometrical	DRAGON
nCp, nCs, nCt, nCq, nCrs, nCrt, nCrq, nCar, nCbH, nCb-, nCconj, nR=Cp, nR=Cs, nR=Ct, nR#CH/X, nR#C-, nRCOOH, nArCOOH, nRCOOR, nArCOOR, nRCONH2, nRCONHR, nArCONHR, nRCONR2, nArCONR2, nROCON, nArOCON, nArCOSR, nRCO, nArCO, nCONN, nN=C-N<, nC(=N)N2, nRC=N, nArC=N, nArCNO, nRNH2, nArNH2, nRNHR, nArNHR, nRNR2, nArNR2, nN-N, nRCN, nArCN nN+, nNq, nRNHO, nArNHO, nArNO2, nN(CO)2, nC=N-N<, nROH, nArOH, nOHp, nOHs, nOHt, nROR, nArOR, nRSR, nSO, nS(=O)2, nSO2OH, nSO3OH, nSO2N, nCH2RX, nR=CRX, nCHRX2, nCRX3, nArX, nCXr, nCXr=, nCconjX, nOxiranes, nAzetidines, nBeta-Lactams, nPyrrolidines, nOxolanes, nPyrroles, nPyrazoles, nImidazoles, nFuranes, nThiophenes, nIsoxazoles, nThiazoles, nTriazoles, nPyridines, nPyridazines, nPyrimidines, nPyrazines, nHDon, nHAcc, nHBonds	Functional Groups	DRAGON
C-001, C-002, C-003, C-004, C-005, C-006, C-007, C-008, C-009, C-010, C-011, C-012, C-013, C-014, C-015, C-016, C-017, C-018, C-019, C-020, C-021, C-022, C-024, C-025, C-026, C-027, C-028, C-029, C-030, C-031, C-032, C-033, C-034, C-035, C-036, C-037, C-038, C-039, C-040, C-041, C-042, C-043, C-044, H-046, H-047, H-048, H-049, H-050, H-051, H-052, H-053, H-054, O-056, O-057, O-058, O-059, O-060, O-061, N-066, N-067, N-068, N-069, N-070, N-071, N-072, N-073, N-074, N-075, N-076, N-077, N-078, N-079, F-081, F-082, F-083, F-084, F-085, Cl-086, Cl-089, Cl-090, Br-091, Br-092, Br-094, S-106, S-107, S-108, S-109, S-110	Atom-centred fragments	DRAGON
Ui, Hy, Hy2, AMR, TPSA(NO), TPSA(Tot), MLOGP, MLOGP2, ALOGP, ALOGP2	Molecular properties	DRAGON
R2_F, R4_F, R2_Cl, R3_Cl, R4_Cl, R2_Br, R4_Br, R2_Me, R3_Me, R4_Me, R5_Me, R2_OH, R4_OH, R6_OH, R2_OMe, R3_OMe, R4_OMe, R5_OMe, R6_OMe, R2_CF3, R3_CF3, R4_CF3, R5_CF3, R4_Et, R3_OCF3, R3_Bn, R5_Bn, R3_NO2, R2_OAc, R5_OAc, R3_CO2Ph, R4_FA026, R4_FA027, R4_FA028, R4_OC6H13, R4_FA029, R5_(4′MPPD), R4_(4′PPZ), R4_(1PPD), R4_(4′EPPD), R4_(4′MPPZ), R4_(4BiPPD), R4_FA031, R4_FA032, R4_FA033, R4_FA030, R2_TMPhO, R′2_F, R′3_F, R′4_F, R′5_F, R′6_F, R′2_Cl, R′3_Cl, R′4_Cl, R′5_Cl, R′2_Br, R′3_Br, R′4_Br, R′5_Br, R′2_Me, R′3_Me, R′4_Me, R′5_Me, R′2_CF3, R′4_CF3, R′2_OH, R′4_OH, R′2_OMe, R′3_OMe, R′4_OMe, R′5_OMe, R′3_NO2, R′4_NO2, R′5_NO2, R′3_CN, R′4_CN, R′3_OC6H13, R′4_SMe, R′4_(4MPPZ), R′4_FB034, R′4_FB035, R′4_nPr, R′3_NH2, R′4_NMe2	Indicative variables	N/A

N/A: None applied.

## Data Availability

Data are contained within the article and Supplementary Materials.

## Supplementary Materials

The following supporting information can be downloaded at https://www.mdpi.com/article/10.3390/molecules28145486/s1. Table S1: List of molecules used for the generation of the QSAR model; Table S2: List of molecules used for the validation test; Table S3: Molecular descriptors for derivatives of compound B and their predicted logIC_50_ values; Figure S1: ^1^H NMR (600 MHz, CDCl_3_) of 2′-hydroxy,4′-cinnamate chalcone (**B**); Figure S2: ^13^C NMR (150 MHz, CDCl_3_) of 2′-hydroxy,4′-cinnamate chalcone (**B**); Figure S3: COSY NMR (600 MHz, CDCl_3_) of 2′-hydroxy,4′-cinnamate chalcone (**B**); Figure S4: HSQC NMR (600 MHz, CDCl_3_) of 2′-hydroxy,4′-cinnamate chalcone (**B**); Figure S5: HMBC NMR (600 MHz, CDCl_3_) of 2′-hydroxy,4′-cinnamate chalcone (**B**); Figure S6: ESI+ mass spectra of 2′-hydroxy,4′-cinnamate chalcone (**B**).

## Sample Availability

Samples of the compounds are available from the authors.
