# Supplementary material for: Finding a Novel Chalcone–Cinnamic Acid Chimeric Compound with Antiproliferative Activity against MCF-7 Cell Line Using a Free-Wilson Type Approach
Source: Molecules. 2023 Jul 18;28(14):5486. doi: 10.3390/molecules28145486 (PMC10383513; doi:10.3390/molecules28145486)
Supplement: Supplementary file 1 [file molecules-28-05486-s001.zip › molecules-2311414-supplementary.pdf]

Finding a Novel Chalcone-Cinnamic Acid Chimeric Compound with Antiproliferative Activity against MCF-7 Cell Line by Means of a Free-Wilson Type Approach

Isis A. Y. Ventura-Salazar [1], Francisco José Palacios-Can [2], Leticia González-Maya[3], Jessica Nayelli Sánchez-Carranza[3], Mayra Antunez-Mojica [4], Rodrigo Said Razo-Hernández [2*] and Laura Alvarez [1*]

[1] Instituto de Investigación en Ciencias Básicas y Aplicadas, Centro de Investigaciones Químicas, Universidad Autónoma del Estado de Morelos, Av. Universidad No. 1001, Cuernavaca 62210, Mor., México. isis.venturaz@uaem.edu.mx, lalvarez@uaem.mx.

[2] Instituto de Investigación en Ciencias Básicas y Aplicadas, Centro de Investigación en Dinámica Celular, Universidad Autónoma del Estado de Morelos, Av. Universidad No. 1001, Cuernavaca 62210, Mor., México. fjpc@uaem.mx, rodrigo.razo@uaem.mx.

[3] Facultad de Farmacia. Universidad Autónoma del Estado de Morelos, Av. Universidad No. 1001, Cuernavaca 62210, Mor., México. jessica.sanchez@uaem.mx, letymaya@uaem.mx.

[4] CONAHCYT-Instituto de Investigación en Ciencias Básicas y Aplicadas, Centro de Investigaciones Químicas, Universidad Autónoma del Estado de Morelos, Av. Universidad No. 1001, Cuernavaca 62210, Mor., México. myam@uaem.mx.

* Correspondence: rodrigo.razo@uaem.mx (R.S.R.H.) and lalvarez@uaem.mx (L.A.)

Corresponding author: Rodrigo Said Razo Hernández email: rodrigo.razo@uaem.mx and Laura Alvarez email: lalvarez@uaem.mx; Tel./Fax: +52-777-329-7997

Citation: Ventura-Salazar, I.A.Y.; Palacios-Can, F.J.; González-Maya, L.; Sánchez-Carranza, J.N.; Antúnez-Mojica, M.; Razo-Hernández, R.S.; Alvarez, L. Finding a Novel Chalcone-Cinnamic Acid Chimeric Compound with Antiproliferative Activity against MCF-7 Cell Line by Means of a Free-Wilson-Type Approach. *Molecules* **2023**, *28*, 5486.

https://doi.org/10.3390/molecules28145486

Academic Editor: Mayra Antúnez-MojicaReceived: date

Received: 14 March 2023
Revised: 30 June 2023
Accepted: 3 July 2023
Published: date

Copyright: © 2023 by the authors. Submitted for possible open access publication under the terms and conditions of the Creative Commons Attribution (CC BY) license (https://creativecommons.org/licenses/by/4.0/).

Content

1. **Table S1**. List of molecules used for the generation of the QSAR model.
2. **Table S2**. List of molecules used for the validation test.
3. **Table S3.** Molecular descriptors for derivatives of compound B and their predicted $logIC_{50}$ values.
4. **Figure S1.** ^{1}H NMR (600 MHz, $CDCl_3$) of 2´-hydroxy,4´-cinnamate chalcone (**B**)
5. **Figure S2.** ^{13}C NMR (150 MHz, $CDCl_3$) of 2´-hydroxy,4´-cinnamate chalcone (**B**)
6. **Figure S3**. COSY NMR (600 MHz, $CDCl_3$) of 2´-hydroxy,4´-cinnamate chalcone (**B**)
7. **Figure S4.** HSQC NMR (600 MHz, $CDCl_3$) of 2´-hydroxy,4´-cinnamate chalcone (**B**)
8. **Figure S5.** HMBC NMR (600 MHz, $CDCl_3$) of 2´-hydroxy,4´-cinnamate chalcone (**B**)
9. **Figure S6.** ESI+ mass spectra of 2´-hydroxy,4´-cinnamate chalcone (**B**).

Table S1. List of molecules used for the generation of the QSAR model. A complete list of their calculated molecular descriptors alongside their experimental values (expressed as the $logIC_{50}$) is shown. Molecules were obtained as follows: From molecules 1 to 18 from reference [1]; 19 to 22 from reference [2]; 23 to 25 from reference [3]; 26 to 31 from reference [4]; 32 to 37 from reference [5]; 38 to 49 from reference [6]; molecule 50 from reference [7]; 51 to 56 from reference [8]; 57 and 58 from reference [9]; 59 to 78 from reference [10]; 79 to 82 from reference [11]; 83 to 104 from reference [12]; 105 to 157 from reference [13]; 158 to 163 from reference [14]; 164 to 174 from reference [15]; 175 to 188 from reference [16]; 189 to 197 from reference [17]; and 198 to 207 from reference [18].

No.	MolID	$logIC_{50}$	R6_OH	R2_OMe	R4_FA026	R4_FA029	R4_(1PPD)	R2_TMPhO	R2′_Cl	R4′_Cl	R2′_OMe	R4′_(4MPPZ)	R4′_FB035	Qindex	CENT	C029	H052
**1**	L019	1.266	0	0	0	0	0	0	0	0	0	0	1	11	786	0	3
**2**	L020	1.303	0	0	0	0	0	0	0	0	0	0	1	12	1005	0	3
**3**	L021	1.205	0	0	0	0	0	0	0	0	0	0	1	14	1439	0	3
**4**	L022	1.275	0	0	0	0	0	0	0	0	0	0	1	15	1216	0	3
**5**	L023	1.279	0	0	0	0	0	0	0	0	0	0	1	14	1052	0	3
**6**	L024	1.195	0	0	0	0	0	0	0	0	0	0	1	15	1160	0	3
**7**	L025	1.131	0	1	0	0	0	0	0	0	0	0	1	12	969	0	3
**8**	L026	1.255	0	0	0	0	0	0	0	0	0	0	1	15	1188	0	3
**9**	L027	1.143	0	0	0	0	0	0	0	0	0	0	1	12	1005	0	3
**10**	L028	1.404	0	0	0	0	0	0	0	0	0	0	1	12	872	0	3
**11**	L029	0.998	0	0	0	0	0	0	0	0	0	0	1	19	1743	0	3
**12**	L030	0.791	0	0	0	0	0	0	0	0	0	0	1	15	1340	0	3
**13**	L031	1.472	0	0	0	0	0	0	0	0	0	0	1	12	987	0	3
**14**	L032	1.404	0	0	0	0	0	0	0	0	0	0	1	12	872	0	3
**15**	L033	1.300	0	0	0	0	0	0	0	0	0	0	1	12	872	0	3
**16**	L034	1.377	0	0	0	0	0	0	0	0	0	0	1	12	872	0	3
**17**	L035	1.313	0	0	0	0	0	0	0	0	0	0	1	12	882	0	3
**18**	L036	1.373	0	0	0	0	0	0	0	0	0	0	1	12	892	0	3
**19**	L039	0.176	0	1	0	0	0	0	0	0	0	0	0	12	668	0	0
**20**	L040	0.491	0	0	0	0	0	0	0	0	0	0	0	12	556	0	0
**21**	L041	-0.149	0	0	0	0	0	0	0	0	0	0	0	13	832	0	0
**22**	L042	0.845	0	0	0	0	0	0	0	1	0	0	0	14	808	0	0

**23**	L058	1.761	0	0	0	0	0	0	0	0	0	0	0	12	470	0	0
**24**	L059	1.761	0	0	0	0	0	0	0	0	0	0	0	12	502	0	0
**25**	L060	1.639	0	0	0	0	0	0	0	0	0	0	0	16	1234	0	0
**26**	L067	1.228	0	0	0	0	0	0	0	0	0	0	0	14	524	0	0
**27**	L068	1.576	0	0	0	0	0	0	0	0	0	0	0	11	344	0	0
**28**	L069	1.158	0	0	0	0	0	0	0	0	0	0	0	11	344	0	0
**29**	L070	1.474	0	0	0	0	0	0	1	0	0	0	0	11	344	0	0
**30**	L071	1.415	0	0	0	0	0	0	0	0	0	0	0	11	352	0	0
**31**	L072	1.530	0	0	0	0	0	0	0	0	0	0	0	11	352	0	0
**32**	L073	0.344	0	0	1	0	0	0	0	0	0	0	0	11	708	0	0
**33**	L074	0.312	0	0	1	0	0	0	0	0	0	0	0	12	904	0	0
**34**	L075	0.513	0	0	1	0	0	0	0	0	0	0	0	12	803	0	0
**35**	L076	2.115	0	0	0	0	0	0	0	0	0	0	0	16	1324	0	0
**36**	L077	1.114	0	0	0	0	0	0	0	0	0	0	0	17	1572	0	0
**37**	L078	1.681	0	0	0	0	0	0	0	0	0	0	0	17	1445	0	0
**38**	L095	1.470	0	0	0	0	0	0	0	0	1	0	0	13	737	0	0
**39**	L096	1.530	0	0	0	0	0	0	0	0	1	0	0	13	816	0	0
**40**	L097	1.519	0	0	0	0	0	0	0	0	1	0	0	13	737	0	0
**41**	L098	1.528	0	0	0	0	0	0	0	0	1	0	0	14	786	0	0
**42**	L099	1.477	0	0	0	0	0	0	0	0	1	0	0	13	737	0	0
**43**	L100	1.213	0	0	0	0	0	0	0	0	1	0	0	15	960	0	0
**44**	L101	1.980	0	0	0	0	0	0	0	0	1	0	0	14	881	0	0
**45**	L102	1.711	0	1	0	0	0	0	0	0	1	0	0	15	1172	0	0
**46**	L103	1.262	0	0	0	0	0	0	0	0	1	0	0	13	737	0	0
**47**	L104	1.367	0	0	0	0	0	0	0	0	1	0	0	22	2730	0	0
**48**	L105	2.261	0	0	0	0	0	0	0	0	1	0	0	13	737	0	0
**49**	L106	1.804	0	0	0	0	0	0	0	0	1	0	0	16	1552	0	0
**50**	L112	2.065	0	0	0	0	0	0	0	0	0	0	0	13	608	0	0
**51**	L113	1.726	0	0	0	0	0	0	0	0	0	0	0	14	1256	0	0
**52**	L114	1.957	0	0	0	0	0	0	0	0	0	0	0	15	1366	0	0

**53**	L115	-0.759	0	0	0	0	0	0	0	0	0	0	0	15	1542	0	0
**54**	L116	1.856	0	0	0	0	0	0	1	0	0	0	0	15	1338	0	0
**55**	L117	-2.174	0	0	0	0	0	0	0	0	0	0	0	15	1352	0	0
**56**	L118	1.965	0	0	0	0	0	0	1	1	0	0	0	16	1523	0	0
**57**	L119	1.620	0	0	0	0	0	0	0	0	0	0	0	12	1188	0	2
**58**	L120	1.615	0	0	0	0	0	0	0	0	0	0	0	11	1080	0	2
**59**	L126	1.520	0	0	0	0	0	0	0	0	0	0	0	9	256	0	0
**60**	L127	1.371	0	0	0	0	0	0	0	0	0	0	0	10	298	0	0
**61**	L128	1.565	0	1	0	0	0	0	0	0	1	0	0	10	348	0	0
**62**	L129	2.004	0	0	0	0	0	0	0	0	0	0	0	10	867	0	2
**63**	L130	1.509	0	0	0	0	0	0	0	0	0	0	0	10	301	0	0
**64**	L131	0.732	0	0	0	0	0	0	0	0	0	0	0	10	352	0	0
**65**	L132	1.605	0	0	0	0	0	0	0	0	0	0	0	10	304	0	0
**66**	L133	1.594	0	0	0	0	0	0	0	0	0	0	0	10	304	0	0
**67**	L134	1.581	0	0	0	0	0	0	0	0	0	0	0	10	356	0	0
**68**	L135	1.594	0	0	0	0	0	0	0	0	0	0	0	11	417	0	0
**69**	L136	1.212	0	0	0	0	0	0	0	0	0	0	0	11	408	0	0
**70**	L137	1.496	0	0	0	0	0	0	0	0	0	0	0	11	1086	0	2
**71**	L138	1.832	0	0	0	0	0	0	0	0	0	0	0	12	470	0	0
**72**	L139	1.212	0	0	0	0	0	0	0	0	1	0	0	11	492	0	0
**73**	L140	1.565	0	0	0	0	0	0	0	0	0	0	0	11	410	0	0
**74**	L141	1.479	0	0	0	0	0	0	0	0	0	0	0	11	476	0	0
**75**	L142	2.201	0	0	0	0	0	0	0	0	0	0	0	11	1016	0	2
**76**	L144	2.251	0	0	0	0	0	0	0	0	0	0	0	13	660	0	0
**77**	L145	1.516	0	0	0	0	0	0	0	0	1	0	0	12	664	0	0
**78**	L146	1.820	0	0	0	0	0	0	0	0	1	0	0	12	572	0	0
**79**	L147	0.924	0	0	0	0	0	0	0	0	0	0	0	11	372	0	0
**80**	L148	1.342	0	0	0	0	0	0	0	0	0	0	0	12	563	0	0
**81**	L149	0.908	0	0	0	0	0	0	0	0	0	0	0	10	317	0	0
**82**	L150	0.724	0	0	0	0	0	0	0	1	0	0	0	12	425	0	0

**83**	L155	1.860	0	0	0	1	0	0	0	0	0	0	0	21	1375	1	0
**84**	L156	1.739	0	0	0	1	0	0	0	0	0	0	0	24	1774	1	0
**85**	L157	1.908	0	0	0	1	0	0	0	0	0	0	0	21	1468	1	0
**86**	L158	1.888	0	0	0	1	0	0	0	0	1	0	0	22	1824	1	0
**87**	L159	0.959	0	0	0	1	0	0	0	0	0	0	0	22	1448	1	0
**88**	L160	1.117	0	0	0	1	0	0	1	1	0	0	0	22	1448	1	0
**89**	L161	1.812	0	0	0	1	0	0	0	0	0	0	0	22	1840	1	0
**90**	L162	1.774	0	0	0	1	0	0	0	0	0	0	0	20	1260	1	0
**91**	L163	1.498	0	0	0	1	0	0	1	0	0	0	0	21	1345	1	0
**92**	L164	1.097	0	0	0	1	0	0	0	1	0	0	0	21	1375	1	0
**93**	L165	1.559	0	0	0	1	0	0	0	0	0	0	0	21	1375	1	0
**94**	L166	1.086	0	0	0	1	0	0	0	0	0	0	0	21	1345	1	0
**95**	L167	1.654	0	0	0	1	0	0	0	0	0	0	0	21	1345	1	0
**96**	L168	1.346	0	0	0	1	0	0	0	0	0	0	0	21	1360	1	0
**97**	L169	1.352	0	0	0	1	0	0	0	0	0	0	0	22	1420	1	0
**98**	L170	1.152	0	0	0	1	0	0	0	0	0	0	0	21	1360	1	0
**99**	L171	1.241	0	0	0	0	0	0	0	0	0	0	0	22	1428	1	0
**100**	L172	1.344	0	0	0	0	0	0	0	0	0	0	0	21	1331	1	0
**101**	L173	1.725	0	0	0	0	0	0	0	0	0	0	0	21	1476	1	0
**102**	L174	1.164	0	0	0	0	0	0	0	0	0	0	0	21	1361	1	0
**103**	L175	1.286	0	0	0	0	0	0	0	0	0	0	0	21	1361	1	0
**104**	L176	1.072	0	0	0	0	0	0	0	0	0	0	0	21	1331	1	0
**105**	L177	0.447	0	1	0	0	0	0	1	0	0	0	0	18	1320	0	4
**106**	L178	0.531	0	1	0	0	0	0	0	0	0	0	0	17	1202	0	4
**107**	L179	0.398	0	1	0	0	0	0	0	1	0	0	0	18	1348	0	4
**108**	L180	0.929	0	1	0	0	0	0	0	0	0	1	0	23	2232	0	4
**109**	L181	0.820	0	1	0	0	0	0	0	0	0	0	0	18	1320	0	4
**110**	L182	0.732	0	1	0	0	0	0	0	0	0	0	0	18	1348	0	4
**111**	L183	0.845	0	1	0	0	0	0	0	0	1	0	0	18	1453	0	4
**112**	L184	0.845	0	1	0	0	0	0	0	0	0	0	0	18	1505	0	4

**113**	L185	0.580	0	1	0	0	0	0	0	0	0	0	0	18	1320	0	4
**114**	L186	0.591	0	1	0	0	0	0	0	0	0	0	0	18	1348	0	4
**115**	L187	0.447	0	1	0	0	0	0	0	0	0	0	0	21	1777	0	4
**116**	L188	0.491	1	1	0	0	0	0	1	0	0	0	0	19	1430	0	4
**117**	L189	0.826	1	1	0	0	0	0	0	1	0	0	0	19	1460	0	4
**118**	L190	0.756	1	1	0	0	0	0	0	0	0	0	0	18	1304	0	4
**119**	L191	0.531	1	1	0	0	0	0	0	0	0	0	0	19	1430	0	4
**120**	L192	0.806	1	1	0	0	0	0	0	0	0	0	0	19	1460	0	4
**121**	L193	1.348	1	1	0	0	0	0	0	0	0	0	0	19	1430	0	4
**122**	L194	0.820	1	1	0	0	0	0	0	0	0	0	0	19	1460	0	4
**123**	L195	1.004	1	1	0	0	0	0	0	0	1	0	0	19	1572	0	4
**124**	L196	1.407	1	1	0	0	0	0	0	0	0	0	0	19	1628	0	4
**125**	L197	1.494	1	1	0	0	0	0	0	0	0	0	0	19	1600	0	4
**126**	L198	1.083	1	1	0	0	0	0	0	0	1	0	0	20	1876	0	4
**127**	L199	0.431	1	1	0	0	0	0	0	0	0	0	0	19	1445	0	4
**128**	L200	1.140	1	1	0	0	0	0	0	0	0	0	0	19	1628	0	4
**129**	L201	1.155	1	1	0	0	0	0	0	0	0	0	0	22	1922	0	4
**130**	L202	1.158	1	1	0	0	0	0	0	0	0	1	0	24	2417	0	4
**131**	L203	1.143	1	1	0	0	0	0	0	0	0	0	0	20	1782	0	4
**132**	L204	1.212	0	0	0	0	0	0	0	0	1	0	0	16	1036	0	0
**133**	L205	1.330	0	0	0	0	0	0	1	0	0	0	0	15	733	0	0
**134**	L206	0.929	0	0	0	0	0	0	0	1	0	0	0	15	751	0	0
**135**	L207	1.699	0	0	0	0	0	0	0	0	0	0	0	14	660	0	0
**136**	L208	1.676	0	0	0	0	1	0	1	0	0	0	0	15	733	0	4
**137**	L209	1.400	0	0	0	0	1	0	0	1	0	0	0	15	751	0	4
**138**	L210	1.699	0	0	0	0	1	0	0	0	0	0	0	14	660	0	4
**139**	L211	1.699	0	0	0	0	1	0	0	0	1	0	0	16	1036	0	4
**140**	L212	0.813	0	1	0	0	0	0	0	1	0	0	0	12	585	0	0
**141**	L213	1.137	0	1	0	0	0	0	0	0	0	1	0	17	1114	0	0
**142**	L214	0.415	0	1	0	0	0	0	1	0	0	0	0	18	1464	0	7

**143**	L215	0.681	0	1	0	0	0	0	0	0	0	0	0	17	1332	0	7
**144**	L216	0.531	0	1	0	0	0	0	0	1	0	0	0	18	1494	0	7
**145**	L217	0.944	0	1	0	0	0	0	0	0	0	1	0	23	2487	0	7
**146**	L218	0.792	0	1	0	0	0	0	1	0	0	0	0	18	1320	0	0
**147**	L219	0.857	0	1	0	0	0	0	0	0	0	0	0	18	1320	0	0
**148**	L220	0.778	0	1	0	0	0	0	0	0	0	0	0	17	1202	0	0
**149**	L221	1.140	0	1	0	0	0	0	0	0	0	1	0	23	2232	0	0
**150**	L222	0.568	0	1	0	0	0	0	1	0	0	0	0	22	2074	0	4
**151**	L223	0.544	0	1	0	0	0	0	0	0	0	0	0	22	2074	0	4
**152**	L224	1.350	0	0	0	0	0	0	1	0	0	0	0	13	559	0	0
**153**	L225	1.072	0	0	0	0	0	0	0	0	1	0	0	13	632	0	0
**154**	L226	1.407	0	0	0	0	0	0	0	0	0	0	0	12	496	0	0
**155**	L227	0.462	0	1	0	0	0	0	1	0	0	0	0	17	1112	0	4
**156**	L228	0.505	0	1	0	0	0	0	0	1	0	0	0	17	1136	0	4
**157**	L229	0.623	0	1	0	0	0	0	0	0	0	0	0	16	1015	0	4
**158**	L230	1.770	0	0	0	0	0	0	0	0	0	0	0	25	1756	1	0
**159**	L231	1.381	0	0	0	0	0	0	0	0	1	0	0	26	2050	1	0
**160**	L232	1.346	0	0	0	0	0	0	0	0	0	0	0	26	1896	1	0
**161**	L233	1.970	0	0	0	0	0	0	0	0	0	0	0	26	1934	1	0
**162**	L234	0.915	0	0	0	0	0	0	0	0	0	0	0	26	2122	1	0
**163**	L235	0.217	0	0	0	0	0	0	0	0	0	0	0	28	2626	1	0
**164**	L236	1.433	0	0	0	0	0	0	0	0	0	0	0	12	590	0	0
**165**	L237	1.452	0	0	0	0	0	0	0	0	0	0	0	13	750	0	0
**166**	L238	1.571	0	0	0	0	0	0	0	0	0	0	0	13	750	0	0
**167**	L239	1.861	0	0	0	0	0	0	0	0	1	0	0	14	873	0	0
**168**	L240	1.450	0	0	0	0	0	0	0	0	0	0	0	14	893	0	0
**169**	L241	1.428	0	0	0	0	0	0	0	0	0	0	0	13	736	0	0
**170**	L242	1.372	0	0	0	0	0	0	0	0	0	0	0	13	640	0	0
**171**	L243	1.237	0	0	0	0	0	0	0	0	0	0	0	13	648	0	0
**172**	L244	1.309	0	0	0	0	0	0	1	0	0	0	0	13	640	0	0

**173**	L245	1.606	0	0	0	0	0	0	0	0	0	0	0	14	814	0	0
**174**	L246	1.234	0	0	0	0	0	0	0	0	0	0	0	14	718	0	0
**175**	L257	0.836	0	0	0	0	0	0	0	0	0	0	0	21	1326	0	0
**176**	L258	0.801	0	0	0	0	0	0	0	0	0	0	0	21	1440	0	0
**177**	L259	0.646	0	0	0	0	0	0	0	0	0	0	0	21	1412	0	0
**178**	L260	0.895	0	0	0	0	0	0	0	0	0	0	0	22	1770	0	0
**179**	L261	0.717	0	0	0	0	0	0	0	0	0	0	0	23	2080	0	0
**180**	L262	0.915	0	0	0	0	0	0	0	0	0	0	0	20	1218	0	0
**181**	L263	0.870	0	0	0	0	0	0	0	1	0	0	0	21	1326	0	0
**182**	L264	0.893	0	0	0	0	0	0	0	0	0	0	0	21	1326	0	0
**183**	L265	1.009	0	0	0	0	0	0	0	0	0	0	0	21	1311	0	0
**184**	L266	0.915	0	0	0	0	0	0	0	0	0	0	0	21	1311	0	0
**185**	L267	0.870	0	0	0	0	0	0	0	0	0	0	0	22	1472	0	0
**186**	L268	0.893	0	0	0	0	0	0	0	0	0	0	0	22	1600	0	0
**187**	L269	1.182	0	0	0	0	0	0	0	1	0	0	0	22	1472	0	0
**188**	L270	1.029	0	0	0	0	0	0	0	0	0	0	0	24	2197	0	0
**189**	L309	0.601	0	0	0	0	0	0	1	1	0	0	0	18	1373	0	0
**190**	L310	1.016	0	0	0	0	0	0	0	0	0	0	0	17	1216	0	0
**191**	L311	0.589	0	0	0	0	0	0	0	0	0	0	0	19	2012	0	0
**192**	L312	0.979	0	0	0	0	0	0	0	0	0	0	0	17	1418	0	0
**193**	L313	1.325	0	0	0	0	0	0	1	0	0	0	0	18	1373	0	0
**194**	L314	0.775	0	0	0	0	0	0	0	0	0	0	0	16	1134	0	0
**195**	L315	1.122	0	0	0	0	0	0	0	1	0	0	0	18	1384	0	0
**196**	L316	1.455	0	0	0	0	0	0	0	0	0	0	0	17	1230	0	0
**197**	L317	0.464	0	0	0	0	0	0	0	0	1	0	0	19	1994	0	0
**198**	L323	0.642	0	0	0	0	0	1	0	0	0	0	0	19	2010	0	0
**199**	L324	0.537	0	0	0	0	0	1	0	1	0	0	0	19	2205	0	0
**200**	L325	0.787	0	0	0	0	0	1	0	0	0	0	0	21	2820	0	0
**201**	L326	0.806	0	0	0	0	0	1	0	0	0	0	0	18	1828	0	0
**202**	L327	0.707	0	0	0	0	0	1	0	0	0	0	0	19	1974	0	0

**203**	L328	0.698	0	0	0	0	0	1	0	0	0	0	0	19	2010	0	0
**204**	L329	0.839	0	0	0	0	0	1	0	0	0	0	0	19	2010	0	0
**205**	L330	0.724	0	0	0	0	0	1	0	0	1	0	0	21	2794	0	0
**206**	L331	0.708	0	0	0	0	0	1	0	0	0	0	0	21	2654	0	0
**207**	L332	0.949	0	0	0	0	0	1	0	0	0	0	0	20	2348	0	0

Table S2. List of molecules used for the validation test. A complete list of their calculated molecular descriptors alongside their predicted $logIC_{50}$ values are shown. Molecules were obtained as follows: molecules 1 to 3 from reference [19]; 4 to 8 from reference [20]; and molecules 9, 10 from reference [21].

No.	MolID	$logIC_{50}$	R6_OH	R2_OMe	R4_FA026	R4_FA029	R4_(1PPD)	R2_TMPhO	R2′_Cl	R4′_Cl	R2′_OMe	R4′_(4MPPZ)	R4′_FB035	Qindex	CENT	C029	H052	$logIC_{50}$ pred
**1**	P001	1.587	1	1	0	0	0	0	0	0	1	0	0	15	1055	0	0	1.44
**2**	P002	1.324	1	1	0	0	0	0	0	0	0	0	0	15	1051	0	0	1.345
**3**	P003	1.858	1	1	0	0	0	0	0	0	1	0	0	15	1045	0	0	1.437
**4**	P004	1.381	1	0	0	0	0	0	0	0	0	0	0	14	880	0	0	1.724
**5**	P005	1.473	1	0	0	0	0	0	0	0	0	0	0	14	987	0	0	1.757
**6**	P006	1.341	1	0	0	0	0	0	0	0	0	0	0	15	1581	0	0	1.85
**7**	P007	1.511	1	0	0	0	0	0	0	0	0	0	0	15	1080	0	2	1.624
**8**	P008	1.771	1	0	0	0	0	0	0	0	0	0	0	17	1728	0	6	1.498
**9**	P009	1.44	0	0	0	0	0	0	0	0	0	0	0	13	565	0	0	1.337
**10**	P010	2.285	1	0	0	0	0	0	0	0	0	0	0	11	352	0	0	1.82

Table S3. Molecular descriptors for derivatives of compound **B** and their predicted $logIC_{50}$ values.

No.	MolID	R6_OH	R2_OMe	R4_FA026	R4_FA029	R4_(1PPD)	R2_TMPhO	R2′_Cl	R4′_Cl	R2′_OMe	R4′_(4MPPZ)	R4′_FB035	Qindex	CENT	C029	H052	$logIC_{50}$ pred
**1**	**B**	1	0	0	0	0	0	0	0	0	0	0	16	1306	0	0	1.6828
**2**	**C**	1	0	0	0	0	0	0	0	0	0	0	21	2332	0	0	1.5671
**3**	**D**	1	0	0	0	0	0	0	0	0	0	0	22	2472	0	0	1.5244
**4**	**E**	1	0	0	0	0	0	0	0	0	0	0	22	2472	0	0	1.5244
**5**	**F**	1	0	0	0	0	0	0	0	0	0	0	22	2472	0	0	1.5244
**6**	**G**	1	0	0	0	0	0	0	0	0	0	0	22	2472	0	0	1.5244
**7**	**H**	1	0	0	0	0	0	0	0	0	0	0	25	3004	0	0	1.4299
**8**	**I**	1	0	0	0	0	0	0	0	0	0	0	22	2634	0	0	1.573
**9**	**J**	1	1	0	0	0	0	0	0	0	0	0	26	3352	0	0	1.1033
**10**	**K**	1	1	0	0	0	0	1	1	0	0	0	28	3856	0	0	0.7705

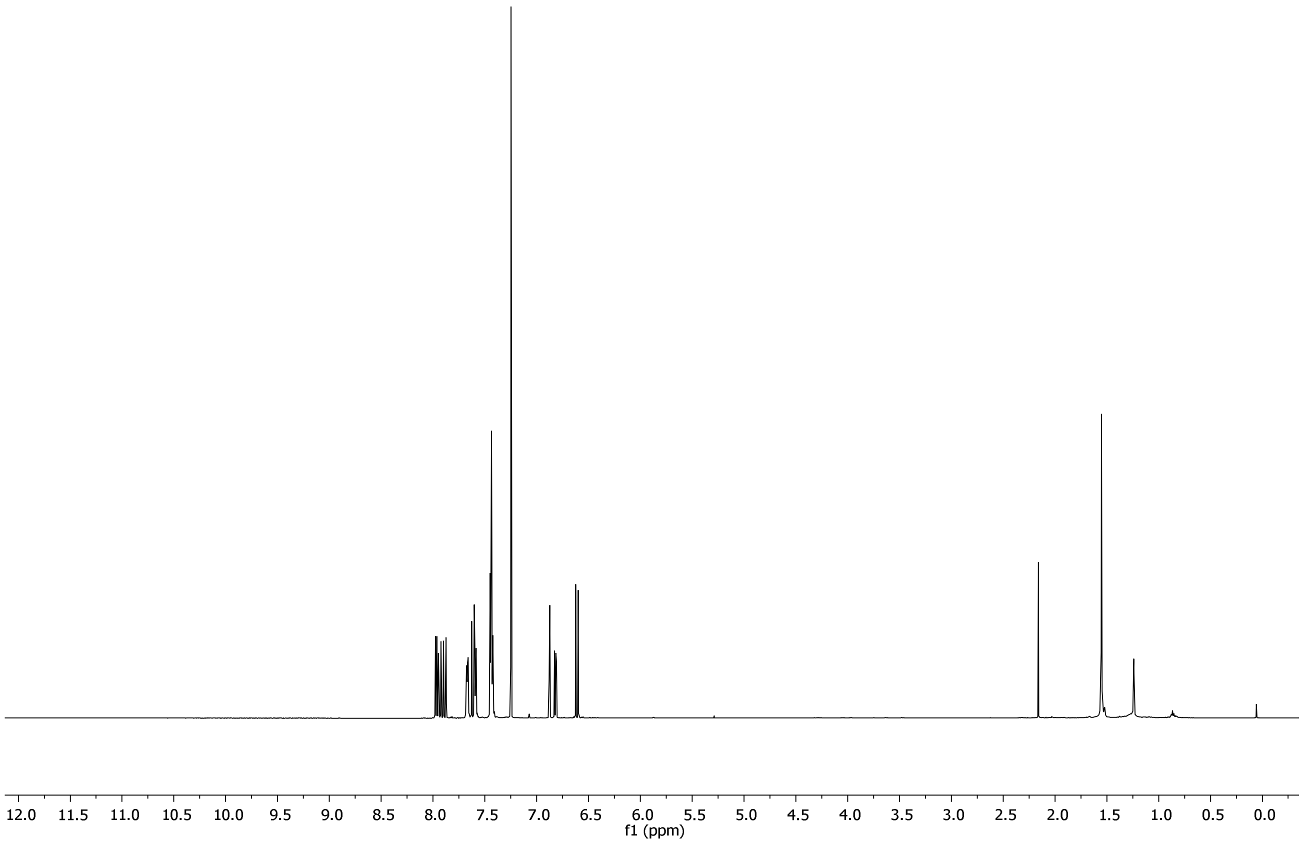

Figure S1. ^{1}H NMR (600 MHz, $CDCl_3$) of 2′-hydroxy,4′-cinnamate chalcone (**B**).

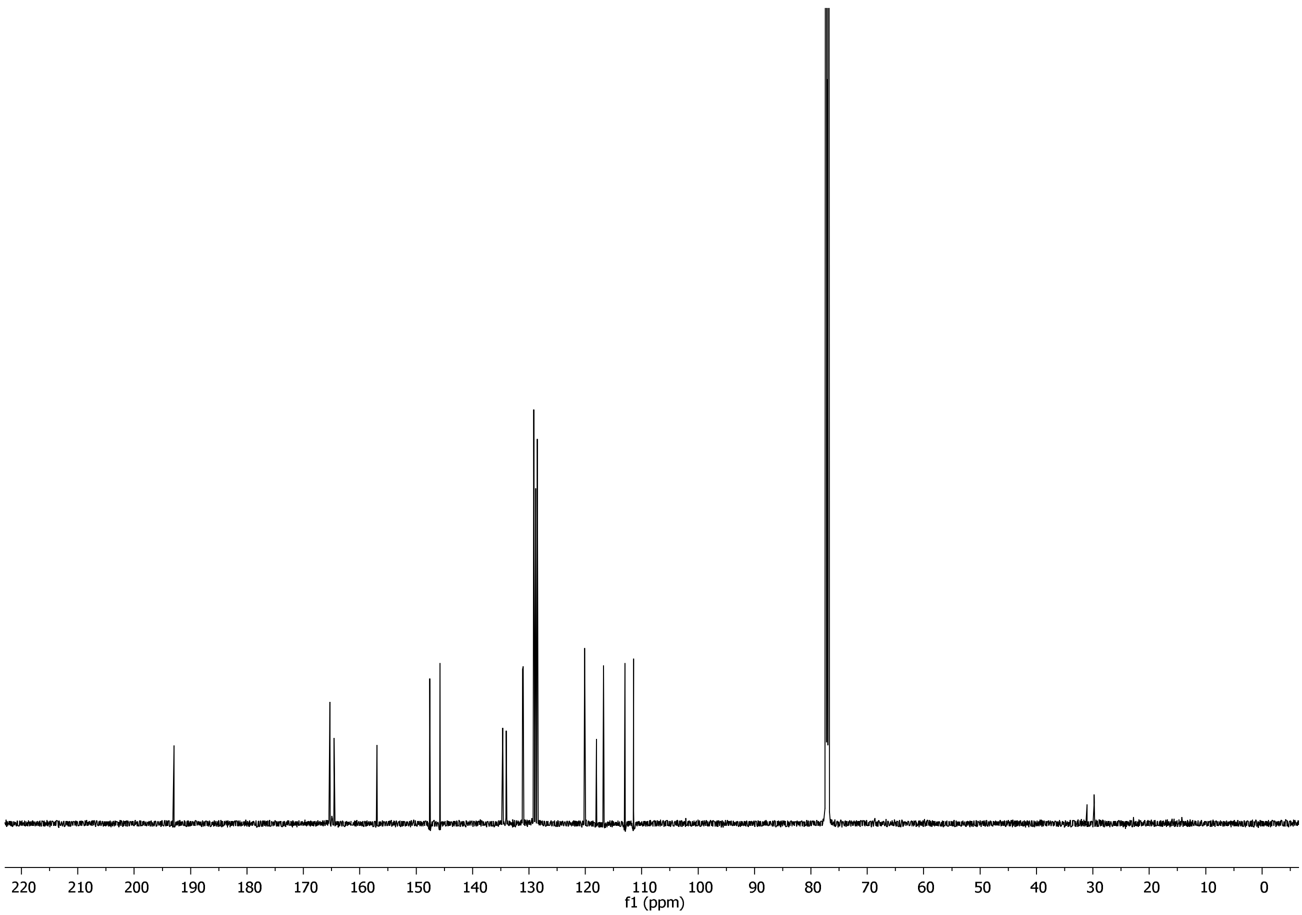

Figure S2. ^{13}C NMR (150 MHz, $CDCl_3$) of 2′-hydroxy,4′-cinnamate chalcone (**B**).

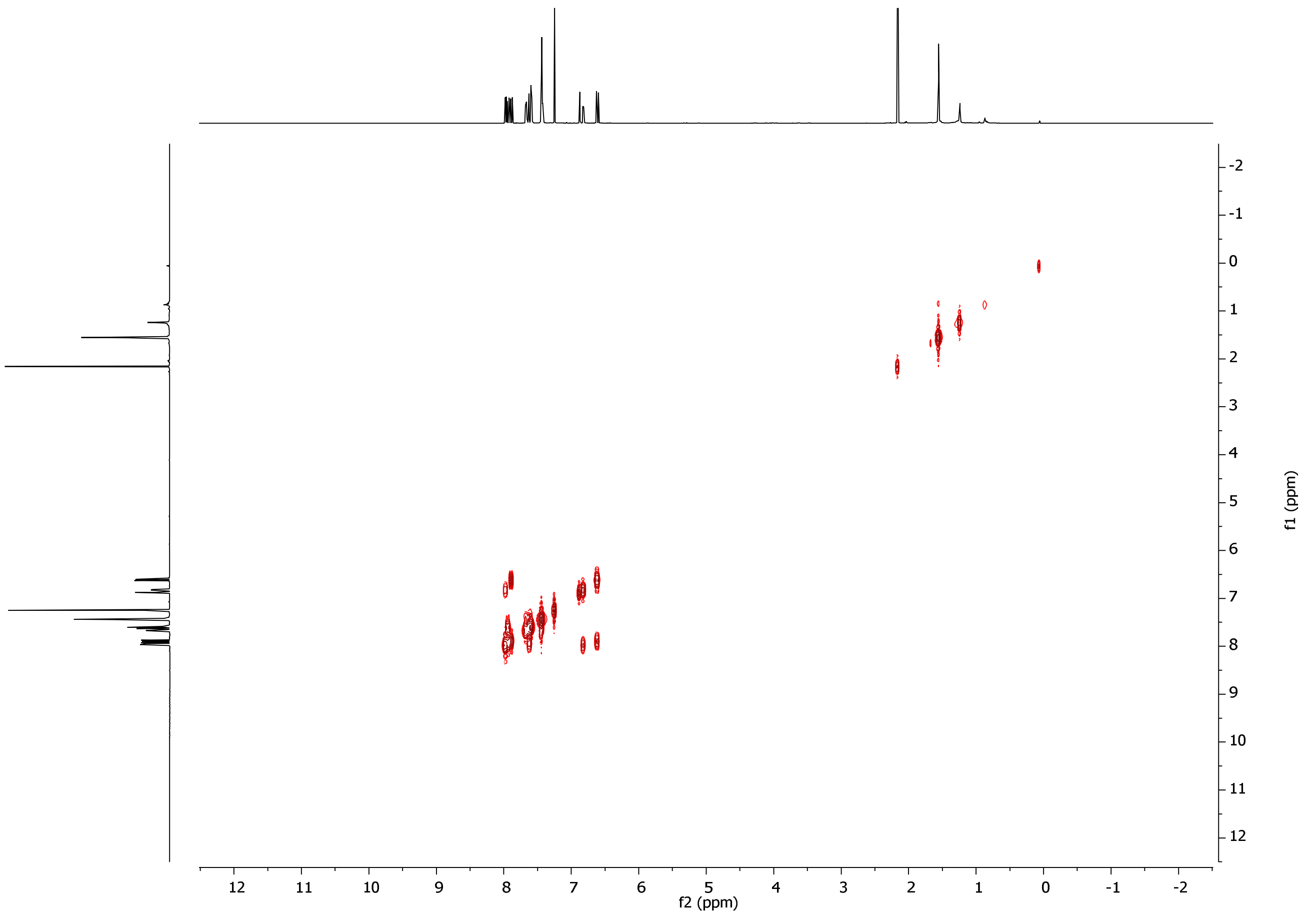

Figure S3. COSY NMR (600 MHz, $CDCl_3$) of 2′-hydroxy,4′-cinnamate chalcone (**B**)

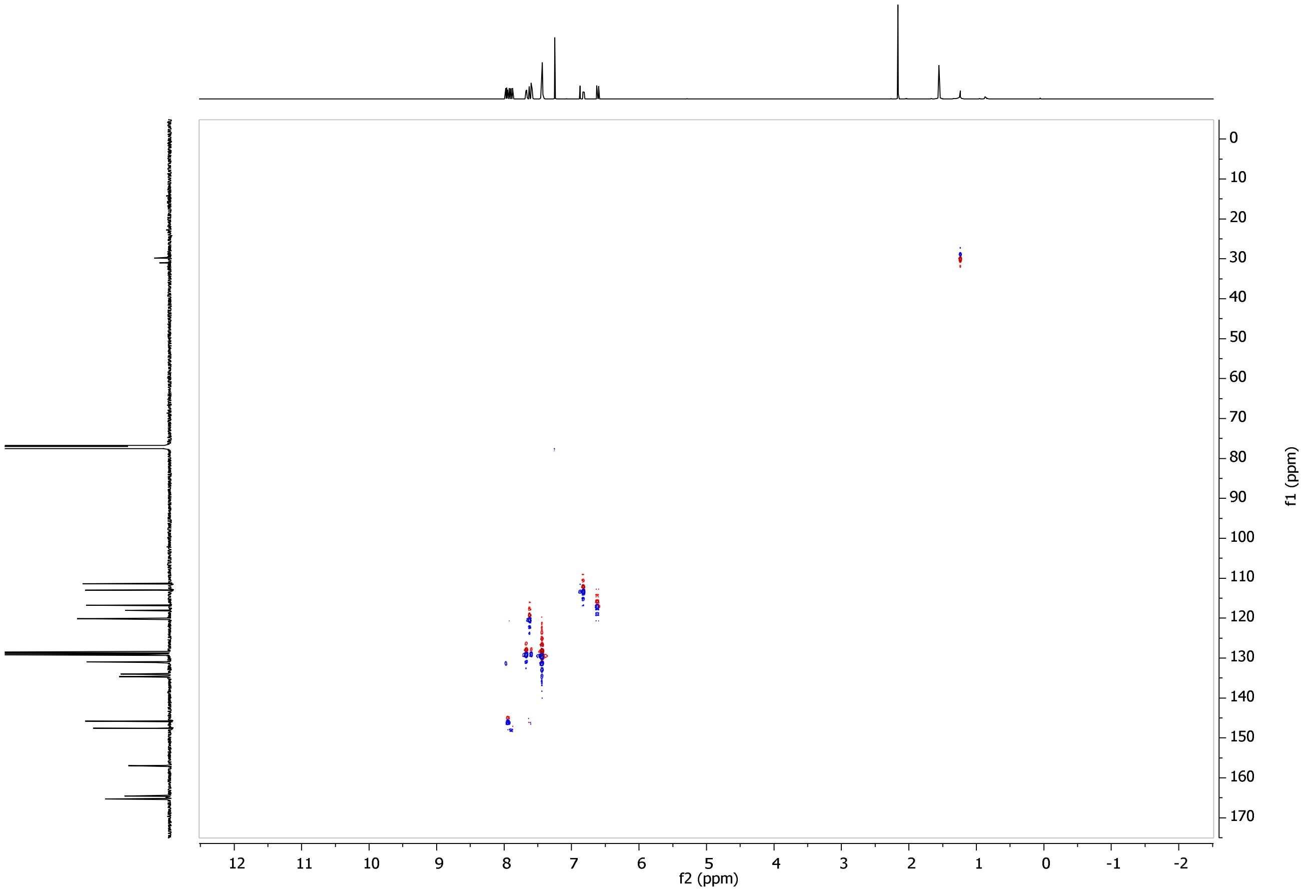

Figure S4. HSQC NMR (600 MHz, $CDCl_3$) of 2′-hydroxy,4′-cinnamate chalcone (**B**)

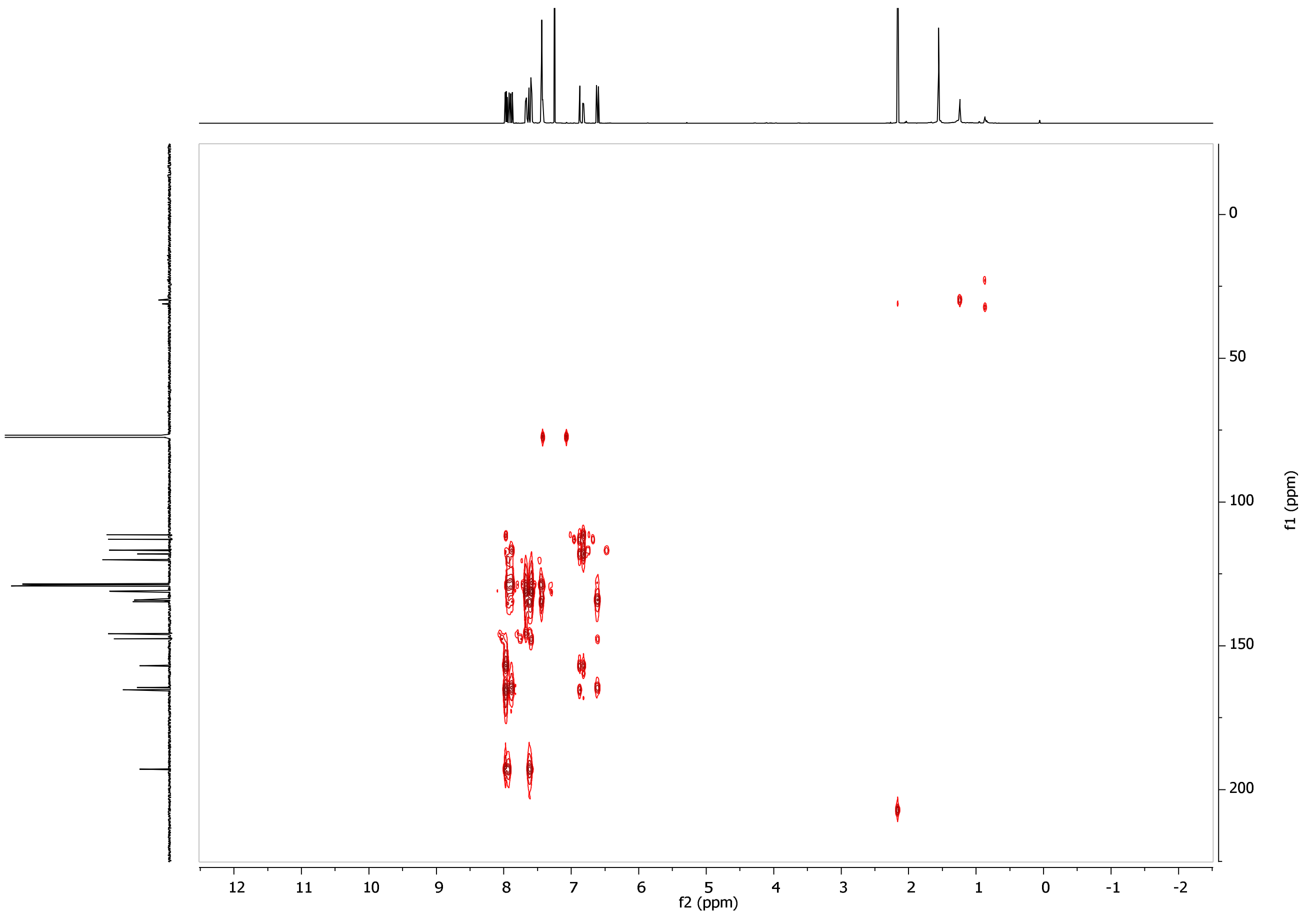

Figure S5. HMBC NMR (600 MHz, CDCl3) of 2′-hydroxy,4′-cinnamate chalcone (B),

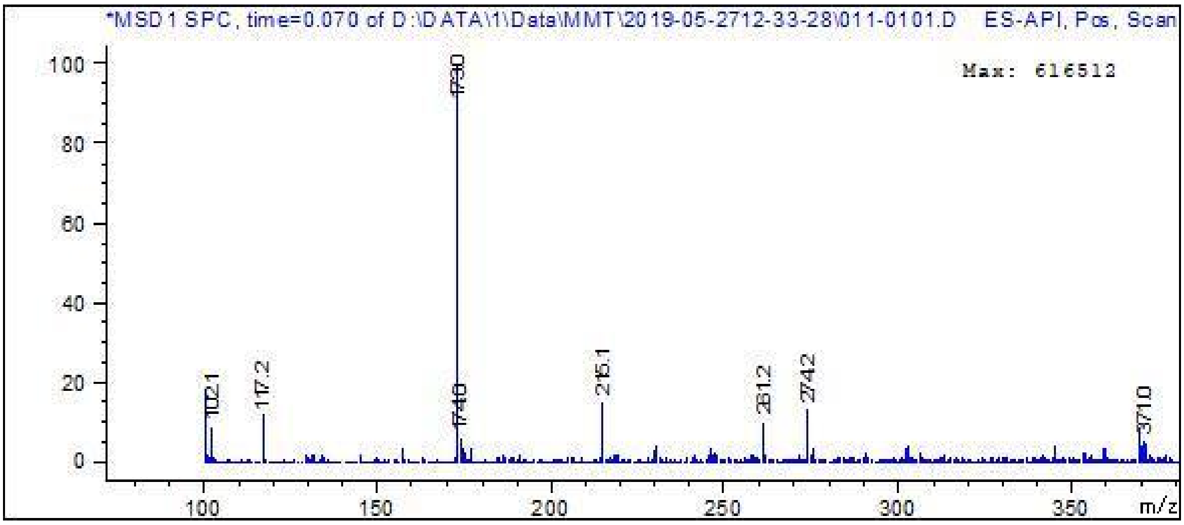

Figure S6. ESI+ mass spectra of 2′-hydroxy,4′-cinnamate chalcone (**B**).

References

1. Qiu, H.-Y.; Wang, F.; Wang, X.; Sun, W.-X.; Qi, J.-L.; Pang, Y.-J.; Yang, R.-W.; Lu, G.-H.; Wang, X.-M.; Yang, Y.-H., Design, Synthesis, and Biological Evaluation of Chalcone-Containing Shikonin Derivatives as Inhibitors of Tubulin Polymerization. ChemMedChem 2017, 12 (5), 399-406. https://doi.org/10.1002/cmdc.201700001
2. Peng, F.; Meng, C.-W.; Zhou, Q.-M.; Chen, J.-P.; Xiong, L., Cytotoxic Evaluation against Breast Cancer Cells of Isoliquiritigenin Analogues from Spatholobus suberectus and Their Synthetic Derivatives. Journal of Natural Products 2016, 79 (1), 248-251. https://doi.org/10.1021/acs.jnatprod.5b00774
3. Sathya, S.; Jonathan, D. R.; Pandiammal, S.; Revathi, B. K.; Usha, G., Synthesis and Screening for Anticancer Activity of a series of Novel Chalcone derivatives on MCF-7 Cell Line. Int. J. ChemTech. Res. 2017, 10 (2), 995-1004. Available online: https://sphinxsai.com/2017/ch_vol10_no2/3/(995-1004)V10N2CT.pdf (accessed on 2 February 2023)
4. Ivković, B. M.; Nikolic, K.; Ilić, B. B.; Žižak, Ž. S.; Novaković, R. B.; Čudina, O. A.; Vladimirov, S. M., Phenylpropiophenone derivatives as potential anticancer agents: Synthesis, biological evaluation and quantitative structure–activity relationship study. European Journal of Medicinal Chemistry 2013, 63, 239-255. https://doi.org/10.1016/j.ejmech.2013.02.013
5. Mohamed, M. F. A.; Shaykoon, M. S. A.; Abdelrahman, M. H.; Elsadek, B. E. M.; Aboraia, A. S.; Abuo-Rahma, G. E.-D. A. A., Design, synthesis, docking studies and biological evaluation of novel chalcone derivatives as potential histone deacetylase inhibitors. Bioorganic Chemistry 2017, 72, 32-41. https://doi.org/10.1016/j.bioorg.2017.03.005
6. Shenvi, S.; Kumar, K.; Hatti, K. S.; Rijesh, K.; Diwakar, L.; Reddy, G. C., Synthesis, anticancer and antioxidant activities of 2,4,5-trimethoxy chalcones and analogues from asaronaldehyde: Structure–activity relationship. European Journal of Medicinal Chemistry 2013, 62, 435-442. https://doi.org/10.1016/j.ejmech.2013.01.018
7. Fikroh, R. A.; Matsjeh, S.; Anwar, C., Synthesis and Anticancer Activity of 2'-hydroxy-2-bromo-4, 5-dimetoxychalcone Against Breast Cancer (MCF-7) Cell Line. Molekul 2020, 15 (1), 34-39. http://dx.doi.org/10.20884/1.jm.2020.15.1.558
8. Durgapal, S. D.; Soni, R.; Umar, S.; Suresh, B.; Soman, S. S., 3-Aminomethyl pyridine chalcone derivatives: Design, synthesis, DNA binding and cytotoxic studies. Chemical Biology & Drug Design 2018, 92 (1), 1279-1287. https://doi.org/10.1111/cbdd.13189
9. Hashim, F.; Wan Mohamed Zin, W. M. K.; Mohamed, M.; Mohd Norhadi Shah, N. S.; Tuan Johari, S. A. T.; Daud, A. I.; Rahamathullah, R., Morphological analysis of MCF-7 cells treated with chalcone derivatives. Frontiers in Pharmacology 2018, 9. https://doi.org/10.3389/conf.fphar.2018.63.00123
10. Mellado, M.; Reyna-Jeldes, M.; Weinstein-Oppenheimer, C.; Coddou, C.; Jara-Gutierrez, C.; Villena, J.; Aguilar, L. F., Inhibition of Caco-2 and MCF-7 cancer cells using chalcones: synthesis, biological evaluation and computational study. Natural Product Research 2022, 36 (17), 4404-4410. https://doi.org/10.1080/14786419.2021.1984465
11. Hussain, A.; Grootveld, M.; Arroo, R.; Beresford, K.; Ruparelia, K.; Fretwell, L., Effects of novel phenolic chalcone derivatives upon MCF-7 Cell viability. Planta Med 2016, 82 (S 01), P929. doi: 10.1055/s-0036-1596927
12. Ma, X.; Wang, D.; Wei, G.; Zhou, Q.; Gan, X., Synthesis and anticancer activity of chalcone–quinoxalin conjugates. Synthetic Communications 2021, 51 (9), 1363-1372. https://doi.org/10.1080/00397911.2021.1881124
13. Liu, X.; Go, M.-L., Antiproliferative activity of chalcones with basic functionalities. Bioorganic & Medicinal Chemistry 2007, 15 (22), 7021-7034. https://doi.org/10.1016/j.bmc.2007.07.042
14. Alswah, M.; Bayoumi, A. H.; Elgamal, K.; Elmorsy, A.; Ihmaid, S.; Ahmed, H. E. A., Design, Synthesis and Cytotoxic Evaluation of Novel Chalcone Derivatives Bearing Triazolo[4,3-a]-quinoxaline Moieties as

Potent Anticancer Agents with Dual EGFR Kinase and Tubulin Polymerization Inhibitory Effects. Molecules 2018, 23 (1), 48. doi:10.3390/molecules23010048
15. Abu Bakar, A.; Akhtar, M. N.; Mohd Ali, N.; Yeap, S. K.; Quah, C. K.; Loh, W.-S.; Alitheen, N. B.; Zareen, S.; Ul-Haq, Z.; Shah, S. A. A., Design, Synthesis and Docking Studies of Flavokawain B Type Chalcones and Their Cytotoxic Effects on MCF-7 and MDA-MB-231 Cell Lines. Molecules 2018, 23 (3), 616. doi:10.3390/molecules23030616
16. Guan, Y.-F.; Liu, X.-J.; Yuan, X.-Y.; Liu, W.-B.; Li, Y.-R.; Yu, G.-X.; Tian, X.-Y.; Zhang, Y.-B.; Song, J.; Li, W.; Zhang, S.-Y., Design, Synthesis, and Anticancer Activity Studies of Novel Quinoline-Chalcone Derivatives. Molecules 2021, 26 (16), 4899. doi:10.3390/molecules26164899
17. Wang, M.; Xu, S.; Wu, C.; Liu, X.; Tao, H.; Huang, Y.; Liu, Y.; Zheng, P.; Zhu, W., Design, synthesis and activity of novel sorafenib analogues bearing chalcone unit. Bioorganic & Medicinal Chemistry Letters 2016, 26 (22), 5450-5454. https://doi.org/10.1016/j.bmcl.2016.10.029
18. Wang, G.; Liu, W.; Gong, Z.; Huang, Y.; Li, Y.; Peng, Z., Design, synthesis, biological evaluation and molecular docking studies of new chalcone derivatives containing diaryl ether moiety as potential anticancer agents and tubulin polymerization inhibitors. Bioorganic Chemistry 2020, 95, 103565. https://doi.org/10.1016/j.bioorg.2019.103565
19. Sangpheak, K.; Mueller, M.; Darai, N.; Wolschann, P.; Suwattanasophon, C.; Ruga, R.; Chavasiri, W.; Seetaha, S.; Choowongkomon, K.; Kungwan, N.; Rungnim, C.; Rungrotmongkol, T., Computational screening of chalcones acting against topoisomerase IIα and their cytotoxicity towards cancer cell lines. Journal of Enzyme Inhibition and Medicinal Chemistry 2019, 34 (1), 134-143. https://doi.org/10.1080/14756366.2018.1507029
20. Xiao, Y.; Lee, I.-S., Effects of Microbial Transformation on the Biological Activities of Prenylated Chalcones from Angelica keiskei. Foods 2022, 11 (4), 543. doi:10.3390/foods11040543
21. Kumar, N.; Kishan, N.; Biswas, S.; Gourishetti, K.; Kamal, M.; Chamallamudi, M. R., Anti-metastatic and Anticancer Potentials of Synthesized Chalcones in B16-F10 Melanoma Cells Induced Metastatic Lung Cancer in C57BL/6 Mice. Indian Journal of Pharmaceutical Education and Research 2021, 55 (3), S742-S750. doi: 10.5530/ijper.55.3s.181
